# An Enzyme‐Like Catalyzed Nanosheets for Redox Stress Oscillation Therapy Against Bacterial Infections

**DOI:** 10.1002/advs.202519334

**Published:** 2025-12-17

**Authors:** Min Ge, Zhao Guo, Zhiming Zhang, Lanlu Lu, Zesong Ruan, Tingwang Shi, Yunfeng Chen, Ju Huang, Chaoliang Tan, Han Lin

**Affiliations:** ^1^ Department of Electrical Engineering City University of Hong Kong 83 Tat Chee Avenue Kowloon Tong Hong Kong SAR 999077 P. R. China; ^2^ Department of Orthopaedics Shanghai Sixth People's Hospital Affiliated to Shanghai Jiao Tong University School of Medicine 600 Yishan Road Shanghai 200233 P. R. China; ^3^ State Key Laboratory of High Performance Ceramics Shanghai Institute of Ceramics Chinese Academy of Sciences; Research Unit of Nanocatalytic Medicine in Specific Therapy for Serious Disease Chinese Academy of Medical Sciences Shanghai 200050 P. R. China; ^4^ National Facility for Protein Science in Shanghai Shanghai Advanced Research Institute Chinese Academy of Sciences Shanghai 201210 P. R. China; ^5^ Department of Ultrasound Women and Children's Hospital of Chongqing Medical University 120 Longshan Road Chongqing 401147 P. R. China

**Keywords:** bacterial metabolism, biofilm eradication, enzyme‐like activity, nanocatalysis, redox regulation

## Abstract

The presence of bacterial biofilms creates a physicochemical barrier, as their dense networks and redox homeostasis prevent the penetration of antimicrobial agents, reactive oxygen species, and host immune cells, rendering them highly resistant to antimicrobial treatment and immune‐mediated killing and clearance. Here, this study demonstrates that SnSe nanosheets with enzyme‐like properties and piezoelectric catalysis can oscillate to regulate bacterial redox homeostasis and improve the lactate‐rich immunosuppressive microenvironment of the infection. This strategy enhances innate immune cell responses to infection or inflammation, achieving effective biofilm clearance on implant surfaces and surrounding tissues in a mouse surgical implant infection model. It reshapes the local immune microenvironment, allowing comprehensive infection control and effective restoration of tissue function. Mechanistically, redox stress oscillation therapy reprograms bacterial amino acid metabolism to induce reductive stress, which then generates oxidative stress under piezoelectric catalysis, resulting in continuously oscillating redox stress within the biofilm. Therefore, this study provides an alternative and promising strategy for the treatment of bacterial biofilm infections with recalcitrant redox homeostasis.

## Graphical abstract

Bacterial biofilms form a physicochemical barrier. Their dense network structure and redox homeostasis confer high resistance to antimicrobial treatments and immune‐mediated killing and clearance. In this study, SnSe nanosheets with enzyme‐like properties and piezoelectric catalysis can oscillate to regulate bacterial redox homeostasis and improve the lactate‐rich immunosuppressive microenvironment of the infection.

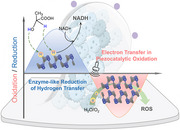

## 1 Introduction

Bacterial infections have become a major challenge to global public health due to the rise of antibiotic‐resistant strains and the persistence of biofilm‐related infections.^[^ ^1^, ^2^ ^]^ With increasing resistance among many pathogens to antibiotics, once considered a rapid and effective means of treating bacterial infections, pathogens such as methicillin‐resistant *Staphylococcus aureus* (MRSA) and multidrug‐resistant Gram‐negative bacteria have become resistant to multiple antibiotics, rendering standard treatments ineffective.^[^ ^3^ ^]^ In addition, bacteria, surrounding cells, the interfacial environment, and extracellular polymers that act as bacterial adhesives form specific biofilms.^[^ ^4^ ^]^ Biofilms act as barriers that prevent the penetration of antimicrobial drugs and immune cells, leading to chronic and recurrent infections.^[^ ^5^ ^]^ In addition, the internal redox homeostasis they form makes them resistant to single external oxidative or reductive stresses,^[^ ^6^, ^7^ ^]^ which greatly limits the efficacy of current treatment strategies, including traditional antimicrobial drugs and active species‐based combination therapies.

In recent decades, physicochemists and biologists have been dedicated to exploring these bioactive substances and their redox chemical reaction mechanisms to advance the development of nanocatalytic and nanoenzyme therapies.^[^ ^8^, ^9^, ^10^, ^11^ ^]^ Significant breakthroughs in nanotechnology have sparked further innovation in catalytic medicine, including the emergence of diverse functional materials with unique radical generation or consumption capabilities.^[^ ^12^, ^13^ ^]^ Among these, our group recently proposed a groundbreaking nanocatalytic therapy against bacterial infections.^[^ ^14^, ^15^, ^16^, ^17^ ^]^ This strategy utilizes nanoscale catalysts to generate reactive oxygen species (ROS) and other intermediates, inducing bacterial apoptosis or ferroptosis‐like death. However, the intrinsic redox homeostasis within biofilms, such as superoxide dismutase,^[^ ^18^ ^]^ catalase,^[^ ^19^ ^]^ and reducing gases like H_2_S,^[^ ^6^ ^]^ counteracts external oxidation by preventing the accumulation of reactive oxidants, significantly reducing the efficacy and dosing safety of these nanocatalytic strategies. While the role of oxidative stress in the adverse effects of nanoparticles has been extensively studied, our understanding of the reductive stress induced by nanomaterials within biofilm redox systems remains limited.^[^ ^20^, ^21^ ^]^ Chen et al. reported that a transition metal boride exhibits dehydrogenase‐like activity, mimicking the enzymatic action of natural dehydrogenases to enhance the concentration of reducing components in key intracellular redox reactions.^[^ ^22^ ^]^ Notably, biofilms exhibit negative feedback regulation in response to either oxidative or reductive stress alone, yet no nanomaterial intervention has achieved oscillatory regulation of biofilm redox homeostasis. Therefore, employing redox pressure oscillation therapy to destabilize persistent biofilm barriers represents a highly promising strategy against bacterial biofilm infections.

Here, we present a conceptually compelling yet barely explored strategy against bacterial biofilms: redox stress oscillatory therapy (ReSOT). This approach leverages the controllable lactate dehydrogenase‐like properties of SnSe nanosheets (NSs) and its piezoelectric catalytic characteristics under ultrasonication (US). By achieving oscillatory regulation of redox attacks through exogenous stimuli, this nanosystem simultaneously modulates bacterial resistance by interfering with endogenous lactate and NADH synthesis. These sequential synergistic effects disrupt the redox homeostasis of the infectious microenvironment, effectively dismantling persistent biofilms. ReSOT induces oscillating redox stress within biofilms through enzyme‐like catalysis‐induced reduction stress followed by piezoelectric catalysis‐mediated oxidation stress. Notably, ReSOT reprograms multiple amino acid metabolic pathways, significantly reducing levels of nicotinamide adenine dinucleotide (NAD) and flavin adenine dinucleotide (FAD), core cofactors for electron transport and energy metabolism, thereby disrupting the coupling efficiency between carbohydrate metabolism and the respiratory chain in bacterial redox homeostasis. Furthermore, ReSOT emerges as a potential strategy to reverse lactic acid‐mediated tolerance in dendritic cell formation, alleviate immunosuppression in the infection microenvironment, and enhance innate immune activity. Thus, this study pioneers an emerging therapeutic pathway for treating refractory implant infections in the post‐antibiotic era, based on specific redox oscillations and immunotherapy.

## 2 Results and Discussion

### 2.1 Synthesis and Characterization of SnSe NSs

**Figure 1:**
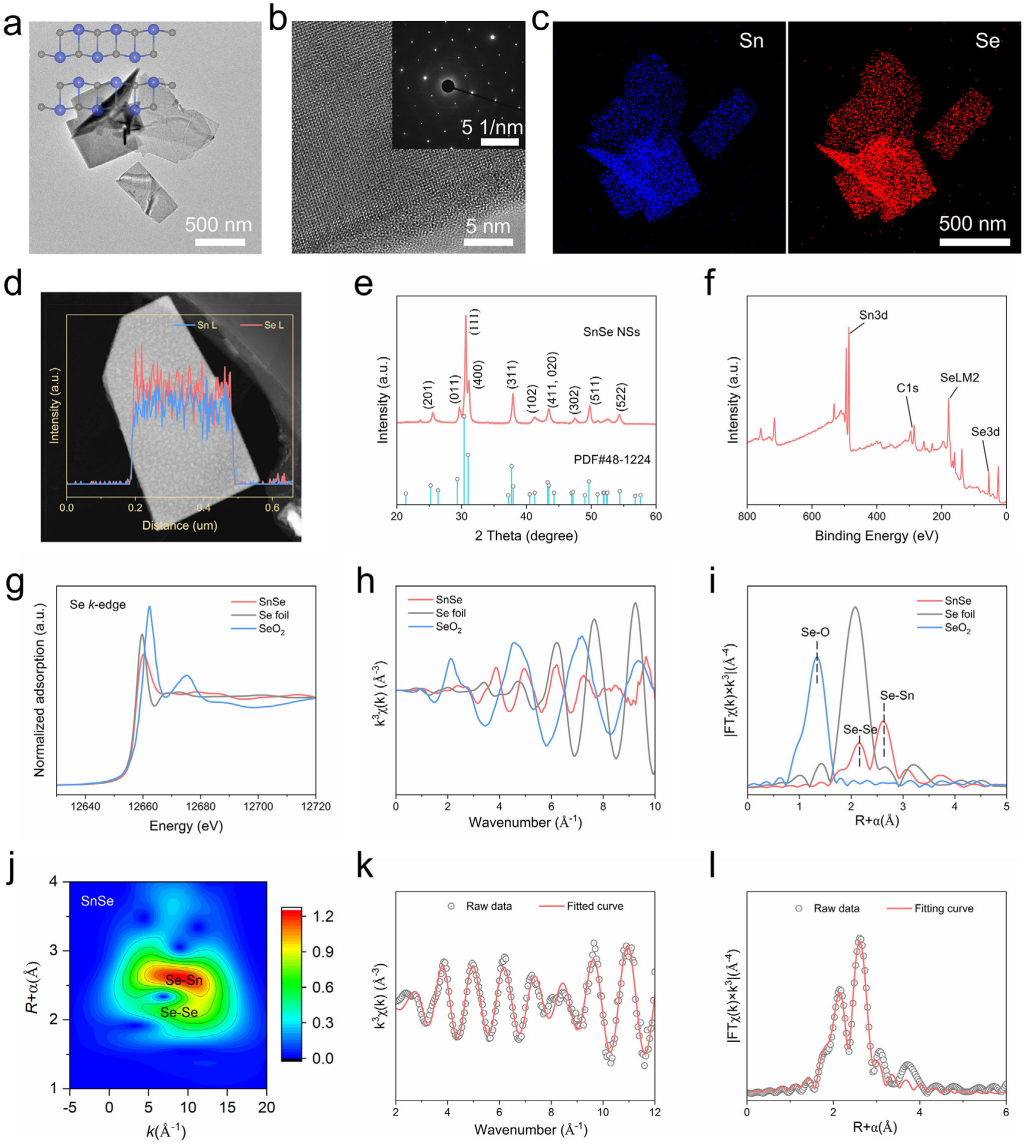
SnSe NSs preparation and characterization. a) TEM image of SnSe NSs and the atomic structure model of b‐axis (inset). Scale bar, 500 nm. b) High‐resolution TEM image and the SAED pattern (inset) of SnSe NSs. Scale bar, 5 nm, 5 1/nm. c) Elemental mapping of SnSe NSs. Scale bar, 500 nm. d) The linear scan element distribution. e) Powder XRD patterns. f) XPS survey spectrum of SnSe NSs. g) Normalized Se k‐edge XANES of SnSe, Se foil, and SeO_2_. h) EXAFS spectra of SnSe, Se foil, and SeO_2_ at k‐space. i) EXAFS spectra of Se k‐edge in the R‐space of SnSe, Se foil, and SeO_2_. j) Wavelet transform images for the EXAFS signals of SnSe NSs. k) The Se k‐edge EXAFS fitting result of SnSe at k space. l) EXAFS fitting result of SnSe in R space.

The 2D SnSe compound was synthesized using a modified co‐precipitation method.^[^ ^23^ ^]^ Transmission electron microscopy (TEM) images showed a typical nanosheet morphology, with lateral dimensions up to several hundred nanometers (**Figure** **1**a). Selected area electron diffraction (SAED) revealed that the SnSe NSs exhibited a typical orthorhombic diffraction pattern (Figure 1b). Combined with powder X‐ray diffraction (XRD) results, the space group was confirmed to be Pnma, corresponding to PDF#48‐1224 (Figure 1e).^[^ ^24^, ^25^ ^]^ The crystal structure of SnSe revealed a distorted atomic arrangement consisting of a strongly bonded double layer dominated by strong Sn─Se bonds in the a‐ and b‐ axis, while weak van der Waals interactions were present along the c‐axis (Figure S1, Supporting Information).^[^ ^26^, ^27^, ^28^ ^]^ The TEM elemental mapping and line profiles demonstrate the uniform distribution of Sn and Se elements within the nanosheets (Figure 1c,d; Figure S2a, Supporting Information). Raman spectroscopy was used to further characterize the local structure of the SnSe NSs.^[^ ^29^ ^]^ As shown in Figure S2b (Supporting Information), SnSe NSs exhibit phonon modes with four characteristic vibrational peaks, and the broad peaks in their Raman spectra indicate the formation of an ultrathin layer structure.^[^ ^30^ ^]^ X‐ray photoelectron spectroscopy (XPS) results show that the Sn:Se molar ratio is close to 1:1, and its chemical properties are further characterized by high‐resolution XPS spectra of the Sn 3d and the Se 3d regions (Figure 1f). The two peaks at 494.5 and 486.1 eV are consistent with the Sn 3d_3/2_ and Sn 3d_5/2_ binding energies of SnSe, and the peaks at 54.7 and 53.8 eV correspond to the Se 3d_3/2_ and Se 3d_5/2_ doublets (Figure S2c,d, Supporting Information).^[^ ^28^, ^31^ ^]^ To further characterize the atomic states and bonding environment in SnSe, we performed X‐ray absorption near‐edge structure (XANES) and extended X‐ray absorption fine structure (EXAFS) studies (Figure 1g–l). The Se k‐edge EXAFS data indicate that the first‐shell R value and the contribution near 2.76 Å (phase uncorrected) in SnSe can be primarily attributed to Se‐Sn coordination.^[^ ^32^ ^]^ The signal near 2.34 Å also suggests a possible Se‐Se contribution, but the coordination number is only 0.2. The high‐quality extended XAFS spectrum enabled us to perform a least‐squares fit of the Se K‐edge data.^[^ ^33^ ^]^ Figure 1i illustrates the coordination environment between SnSe NSs. We also performed a high‐resolution wavelet transform (WT) analysis of the Se k‐edge oscillations (Figure 1j). The quantitative results are summarized in Table S1 (Supporting Information). The results show an R factor of 0.0055 for the synthesized SnSe NSs, confirming the good agreement between the fitted results and the experimental data in Figure 1k,l and Figures S3 and S4 (Supporting Information). Due to the larger Sn─Se bond length, the SnSe NSs product promotes the localization of more electrons on Se atoms, thereby facilitating H adsorption on negatively charged Se anions, which provides potential for H transfer.^[^ ^34^ ^]^

### 2.2 Properties and Mechanisms of SnSe‐Mediated Redox Stress Oscillation

**Figure 2:**
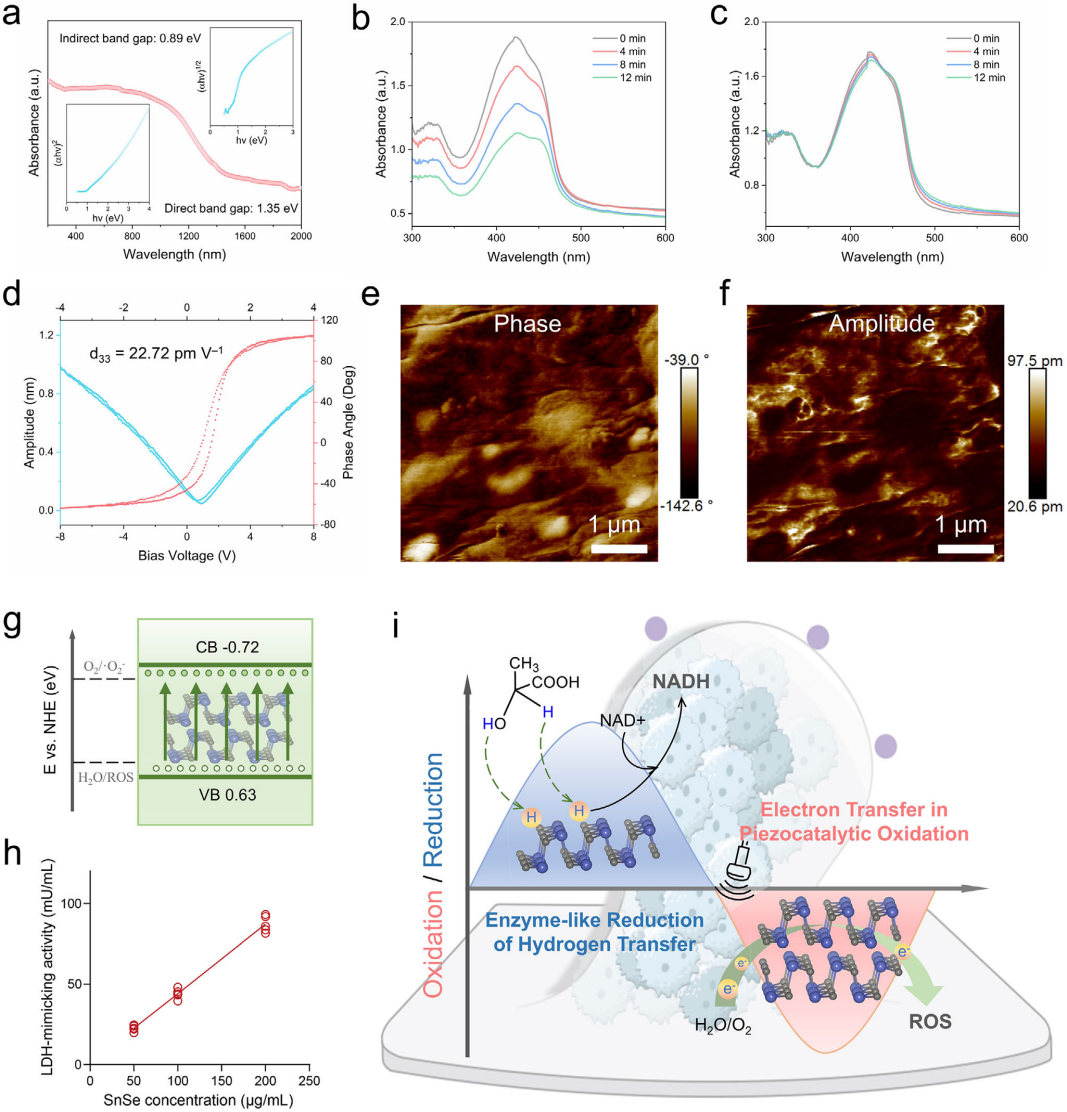
Properties and mechanism of redox stress oscillation therapy. a) UV–vis–NIR diffuse reflectance spectra and tauc plots of SnSe NSs. b) Piezoelectric catalytic degradation efficiency of DPBF under different US times. c) Degradation efficiency of DPBF by US alone at different times. d) Piezoresponsive amplitude curve and phase curve of SnSe NSs. e) Corresponding PFM phase and f) amplitude images. Scale bar, 1 µm. g) Schematic diagram of band energy structure. h) Lactate dehydrogenase‐mimicking activities of SnSe NSs. i) Schematic diagram of redox stress oscillation therapy.

To investigate the catalytic potential of SnSe, we measured their optical bandgaps using UV–vis–NIR diffuse reflectance spectroscopy (**Figure** **2**a). The optical absorption spectra and Tauc plots of the Kubelka‐Munk function for SnSe NSs reveal direct and indirect bandgaps of 1.35 and 0.89 eV, respectively, which are comparable to the bandgap values reported for 2D SnSe materials.^[^ ^28^ ^]^ Next, to assess the ROS‐catalyzing performance of SnSe NSs, we employed 1,3‐diphenylisobenzofuran (DPBF) as a probe. Under different US durations, DPBF underwent a Diels‐Alder 1,4‐cycloaddition reaction with ROS. The decrease in DPBF's absorption intensity at 420 nm indicated ROS generation. To avoid potential photolytic interference, the entire catalytic degradation process was conducted in the dark. As anticipated, the fluorescence intensity of DPBF added to SnSe decreased significantly with US time (Figure 2b), whereas the US‐only group exhibited a negligible change (Figure 2c). Furthermore, to explore the mechanism of ROS generation catalyzed by SnSe NSs, we tested their piezoelectric effect using piezoelectric force microscopy. The typical butterfly‐shaped amplitude curve of SnSe indicates that varying the applied electric field induces strain in the material, exhibiting polarization reversibility (Figure 2d–f).^[^ ^35^, ^36^ ^]^ The d_33_ of SnSe was calculated from the amplitude‐voltage loop to be 22.72 pm V^−1^ (Figure S5a, Supporting Information). Combining the direct bandgap of 1.35 eV estimated from DRS spectroscopy and the valence band edge value (Figure S5b, Supporting Information), the conduction band edge of SnSe NSs is estimated to be approximately −0.72 eV, and the corresponding band structure and piezoelectric catalytic electron transfer are shown in Figure 2g. Additionally, the enzyme‐like activity of SnSe NSs was determined using a lactate dehydrogenase assay kit. The results showed that the catalytic efficiency of SnSe's reductase‐like activity exhibited linear concentration dependence within the measurement range, reaching 44.02 mU/mL at 100 ppm (Figure 2h). This indicates that SnSe effectively catalyzes the reduction of lactate to pyruvate, while the removed hydrogen atoms can transfer to NAD⁺ to form NADH.^[^ ^22^, ^24^ ^]^ In summary, the aforementioned enzyme‐like reduction and catalytic oxidation properties of SnSe NSs provide potential for redox oscillation therapy (Figure 2i).

### 2.3 SnSe‐Mediated ReSOT for Potent Biofilm Eradication

**Figure 3:**
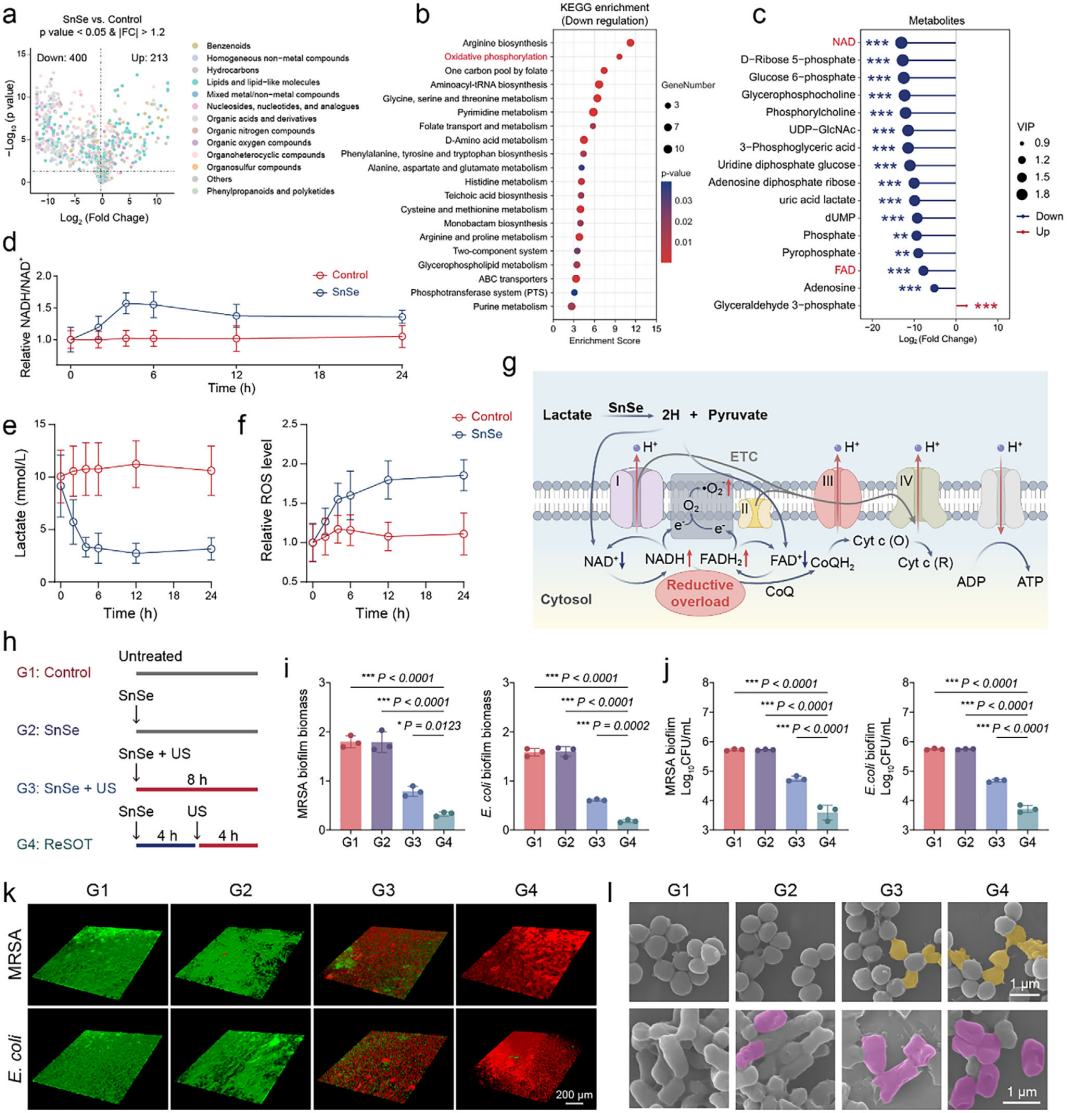
SnSe‐Mediated ReSOT for Potent Biofilm Eradication. a) Volcano plot of differential metabolites after SnSe intervention. b) KEGG enrichment of differential metabolites after SnSe intervention. c) Lollipop plot in carbohydrate metabolism‐related metabolites after SnSe intervention. d) Time‐course of intracellular NADH/NAD⁺ ratio in MRSA biofilm following SnSe intervention, *n* = 6. e) Time‐course of lactate concentration in MRSA biofilm supernatant following SnSe intervention, *n* = 6. f) Time‐course of intracellular ROS level in MRSA biofilm following SnSe intervention, *n* = 6. g) Schematic diagram of SnSe inducing reductive stress in bacteria. h) Schematic of the ReSOT treatment strategy. i) Biofilm biomass of MRSA and *E. coli* biofilms identified by crystal violet staining after various treatments, *n* = 3. j) Quantitative analysis of SPC assays from MRSA and *E. coli* biofilms after various treatments, *n* = 3. k) Representative confocal microscopy images of biofilms from MRSA and *E. coli* after various treatments. Scale bar, 200 µm. l) Representative SEM images of MRSA and *E. coli* after various treatments. The yellow pseudocolor represents damaged MRSA, and the magenta pseudocolor represents damaged *E. coli*. Scale bar, 1 µm. ^*^ *p* < 0.05, ^**^ *p* < 0.01, ^***^ *p* < 0.001. Data are means ± SD.

Bacterial biofilms rely on an extracellular polymeric matrix to establish a stable protective microenvironment, which markedly enhances bacterial tolerance to both antimicrobial agents and host immune responses.^[^ ^4^, ^37^ ^]^ Notably, metabolic heterogeneity within biofilms is particularly pronounced, with excessive lactate accumulation not only maintaining an acidic microenvironment but also promoting bacterial tolerance and immune evasion.^[^ ^38^, ^39^ ^]^ Therefore, targeting lactate metabolism is considered a potential strategy to disrupt biofilm homeostasis. We quantified lactate levels in both mature biofilms formed under static culture and planktonic bacteria grown under continuous shaking. Biofilms exhibited markedly elevated lactate concentrations, whereas lactate levels in planktonic bacteria remained relatively low, indicating that mature biofilms feature a high‐lactate microenvironment (Figure S6, Supporting Information). Given the excellent lactate dehydrogenase‐mimicking activity of our SnSe nanoplatform, it holds potential to modulate lactate levels within the biofilm microenvironment, providing a novel strategy to disrupt biofilm integrity. We first evaluated the effect of SnSe on cell viability using the CCK‐8 assay to determine a safe working concentration. SnSe showed negligible cytotoxicity across the concentration range of 0–600 µg mL^−1^ (Figure S7, Supporting Information). Accordingly, a concentration of 100 µg mL^−1^ was chosen for subsequent evaluation of its anti‐biofilm activity. To systematically evaluate the effects of SnSe on bacterial metabolism, untargeted metabolomic analysis was performed on MRSA (ATCC 43 300). The results revealed widespread metabolic perturbations within the MRSA upon SnSe treatment, including a significant decrease in 400 metabolites and a significant increase in 213 metabolites (**Figure** **3**a). Principal component analysis showed clear separation between the control and SnSe‐treated groups in the principal component space, indicating that SnSe induced consistent and significant changes in the overall metabolic profile of the samples (Figure S8, Supporting Information). Kyoto Encyclopedia of Genes and Genomes (KEGG) pathway enrichment analysis revealed that SnSe treatment markedly suppressed multiple amino acid metabolism pathways (arginine, glycine, serine, etc.), folate‐mediated one‐carbon metabolism, and oxidative phosphorylation, suggesting disruption of bacterial energy homeostasis (Figure 3b). Further analysis of the significantly altered metabolites revealed notable changes in molecules closely associated with carbohydrate metabolism. Key glycolytic intermediates, such as glucose 6‐phosphate and 3‐phosphoglyceric acid, were significantly reduced, indicating disturbed glycolytic flux. Meanwhile, the levels of nucleotide sugars, including Uridine diphosphate (UDP) glucose and UDP‐N‐acetylglucosamine (UDP‐GlcNAc), were reduced, reflecting impaired glucose activation and extracellular polysaccharide biosynthesis. The decrease in D‐ribose‐5‐phosphate suggested disruption of the pentose phosphate pathway, thereby limiting NADPH supply and nucleotide precursor synthesis. Importantly, the levels of NAD and FAD, core cofactors in electron transport and energy metabolism, were significantly decreased, indicating that bacterial redox balance was disturbed and the coupling efficiency between carbohydrate metabolism and the respiratory chain was compromised (Figure 3c). Considering the excellent lactate dehydrogenase‐mimicking activity of SnSe demonstrated in abiotic assays, which catalyzes the oxidation of lactate to pyruvate accompanied by the reduction of NAD⁺ to NADH, the observed decrease in NAD levels may result from its continuous consumption during the SnSe‐driven lactate dehydrogenation reaction. Based on this, we further monitored the dynamic changes of intracellular NADH/NAD⁺ in MRSA following SnSe intervention. The results showed that SnSe increased the NADH/NAD⁺ ratio in MRSA, exhibiting a continuous upward trend (Figure 3d). Consistently, lactate concentrations in the biofilm supernatant steadily decreased after SnSe intervention (Figure 3e). Notably, SnSe also led to a sustained increase in intracellular ROS levels (Figure 3f). Inspired by previous studies,^[^ ^22^ ^]^ the above observations suggest that SnSe intervention may induce a reductive stress state in MRSA.

Specifically, SnSe catalyzes the removal of two hydrogen atoms from a lactate molecule, oxidizing it to pyruvate, while the removed hydrogens are transferred to NAD⁺ to form NADH. This catalytic process leads to substantial consumption of intracellular NAD⁺ and significant accumulation of NADH. The excess NADH then causes over‐reduction of membrane‐bound electron transport chain complexes, including flavin mononucleotide and FAD in Complexes I and II, as well as the semiquinone intermediates of coenzyme Q in Complex III. Excess electrons exceed the normal transfer capacity of these complexes, resulting in an overloaded reduction pool and partial electron retention at membrane‐bound sites. This retention of electrons directly increases the probability of electron leakage. And these leaked electrons can be captured by molecular oxygen to generate superoxide radicals (·O_2_^−^), further disrupting the cellular redox balance and promoting the accumulation of ROS in MRSA (Figure 3g). Based on the above experiments, we propose that SnSe can induce reductive stress in bacteria in the absence of US stimulation. Given its excellent piezoelectric catalytic activity for ROS generation under US, we developed a novel redox stress oscillation therapy, for treating bacterial biofilms. Specifically, bacteria are initially driven into a reductive stress state by SnSe, followed by US activation of the SnSe piezoelectric effect to catalytically generate abundant ROS, thereby inducing a strong oxidative stress state in the bacteria (Figure 3h). Compared with SnSe + US treatment, ReSOT induced a higher level of ROS in MRSA, indicating that ReSOT inflicts more pronounced oxidative damage to the bacteria (Figure S9, Supporting Information). Antibiofilm property of ReSOT was evaluated using MRSA and *Escherichia coli* (*E. coli*, ATCC 35 218) biofilms. Crystal violet staining revealed that ReSOT treatment significantly reduced biofilm biomass and exhibited a stronger inhibitory effect compared to the SnSe + US treatment (Figure 3i; Figure S10, Supporting Information). To further assess the effect of ReSOT on biofilm extracellular polymeric substances (EPS), we measured EPS polysaccharide content. The ReSOT group showed a significant reduction compared with the control group, but a level comparable to the SnSe + US group. This similarity may reflect that, for this specific polysaccharide‐related endpoint, both ReSOT‐induced redox oscillation and SnSe + US‐induced oxidative stress can effectively impair polysaccharide synthesis and stability, resulting in comparable outcomes (Figure S11, Supporting Information). Further standard plate count (SPC) assays showed that ReSOT displayed a clear advantage in killing biofilm‐embedded bacteria, with the number of viable bacteria in the treated group significantly lower than in all control groups (Figure 3j; Figure S12, Supporting Information). To determine whether the US parameters used in this study independently affect biofilm integrity, an US‐only control group was included. The results showed that US alone did not cause significant changes in biofilm structure or biomass (Figure S13, Supporting Information). These findings, together with the original data, indicate that the observed biofilm disruption is primarily attributable to the piezoelectric response of SnSe rather than the ultrasound itself. To further evaluate the contribution of ROS‐mediated oxidative damage to the antibacterial activity of ReSOT, we introduced an ROS scavenger (ascorbic acid) during treatment. The presence of ascorbic acid markedly reduced the antibacterial efficacy of ReSOT, indicating that ROS generation is essential for ReSOT‐induced biofilm disruption and bacterial killing (Figure S14, Supporting Information). Confocal microscopy observation demonstrated that ReSOT disrupted biofilm structure and drastically reduced the number of live bacteria within the biofilm (Figure 3k). Scanning electron microscope (SEM) further revealed that under ReSOT treatment, bacterial cell membranes were severely damaged and cell morphology was markedly collapsed (Figure 3l).

Notably, although SnSe alone could increase intracellular ROS in bacteria, it did not exhibit a significant antibacterial effect. This is probably due to limitations in ROS type (·O_2_^−^), concentration, and subcellular localization, which are insufficient to overcome the bacteria's intrinsic antioxidant defense systems, such as superoxide dismutase. In summary, ReSOT reprograms bacterial metabolism to induce reductive stress, followed by US‐activated piezoelectric catalysis that generates abundant oxidative stress, thereby imposing sequential redox pressures within the biofilm. This strategy efficiently disrupts biofilm structure, compromises bacterial membranes, and achieves potent bacterial eradication, highlighting its promise as a novel anti‐biofilm therapy.

### 2.4 SnSe Reverses Lactate‐Mediated Immunosuppression and Restores Innate Immune Cell Function

**Figure 4:**
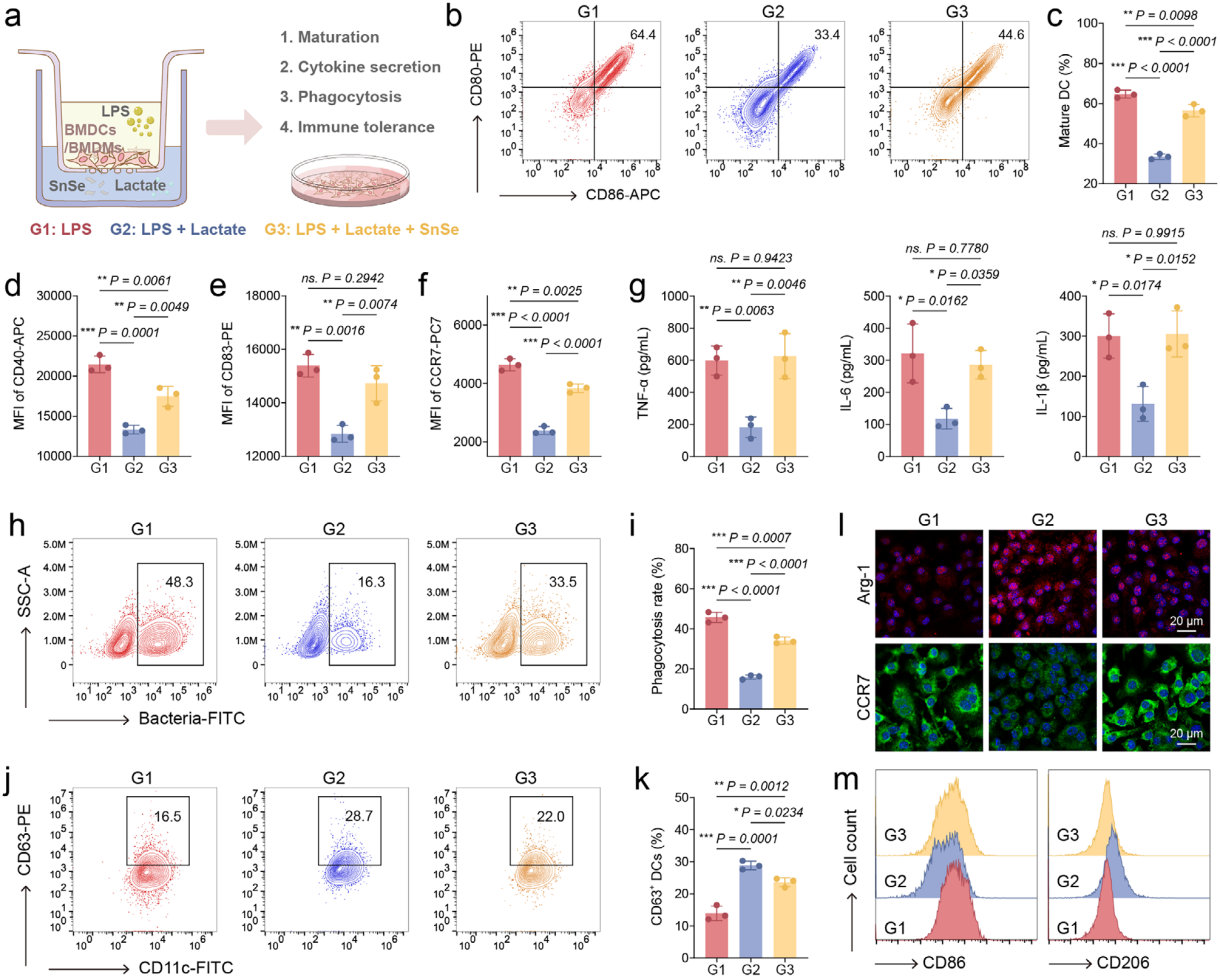
SnSe reverses lactate‐mediated immunosuppression and restores innate immune cell function. a) Schematic diagram of experimental workflow. b) Flow cytometric analysis and c) corresponding quantitative analysis of CD11c^+^CD80^+^CD86^+^ mature DCs, *n* = 3. d) Quantitative analysis of CD40 mean fluorescence intensity (MFI) detected by flow cytometry, *n* = 3. e) Quantitative analysis of CD83 MFI detected by flow cytometry, *n* = 3. f) Quantitative analysis of CCR7 MFI detected by flow cytometry, *n* = 3. g) TNF‐α, IL‐6 and IL‐1β secreted by BMDCs detected by ELISA, *n* = 3. h) Phagocytosis rate and i) corresponding quantitative analysis of BMDCs after various treatment, *n* = 3. j) Flow cytometric analysis and k) corresponding quantitative analysis of CD11c^+^CD63^+^ tDCs, *n* = 3. l) Representative confocal images of expression of CCR7 and Arg‐1 of BMDMs after various treatments. Scale bar, 50 µm. m) Flow cytometric analysis of CD86 and CD206 expression in BMDMs after various treatments. ^*^ *p* < 0.05, ^**^ *p* < 0.01, ^***^ *p* < 0.001. Data are means ± SD.

In the biofilm infection microenvironment, excessive lactate accumulation profoundly suppresses host immunity.^[^ ^39^ ^]^ High lactate levels compromise the phagocytic activity of neutrophils, macrophages, and dendritic cells, inhibit proinflammatory cytokine secretion, and impair T cell proliferation and cytotoxic activity, collectively weakening host defense.^[^ ^40^, ^41^, ^42^, ^43^, ^44^ ^]^ Given these multifaceted immunosuppressive effects, targeting lactate metabolism to reduce its accumulation offers a promising strategy to restore immune competence against biofilm infections. Based on this rationale, we next evaluated whether the lactate dehydrogenase‐mimicking activity of SnSe could rescue immune cell functions, including those of bone marrow‐derived dendritic cells (BMDCs) and bone marrow‐derived macrophages (BMDMs) under lipopolysaccharide (LPS) stimulation (**Figure** **4**a).

Dendritic cells (DCs), as key antigen‐presenting cells, not only capture and process pathogens in innate immunity but also transmit information to T cells through co‐stimulatory molecules, such as CD80 and CD86, and chemokine receptors, such as CCR7, thereby bridging innate and adaptive immunity.^[^ ^45^, ^46^ ^]^ Flow cytometry analysis showed that lactate suppressed DC maturation under LPS exposure, whereas SnSe markedly reversed this effect, leading to a significant increase in the proportion of CD80⁺CD86⁺ mature DCs (Figure 4b,c). In addition, the expression levels of two other co‐stimulatory molecules, CD40 and CD83, were also notably restored by SnSe treatment (Figure 4d,e; Figure S15a,b, Supporting Information). CCR7, a key chemokine receptor on the DC surface, binds to CCL21 in lymphatic vessels to guide DC migration from infected tissues to lymph nodes, a critical step for T cell activation and initiation of adaptive immune responses.^[^ ^47^ ^]^ Flow cytometry results revealed that lactate significantly inhibited CCR7 expression on DCs, whereas SnSe effectively reversed this suppression (Figure 4f; Figure S15c, Supporting Information), suggesting a role for SnSe in restoring DC migratory capacity within an immunosuppressive microenvironment. Moreover, enzyme‐linked immunosorbent assay (ELISA) results demonstrated that SnSe restored the secretion of proinflammatory cytokines TNF‐α, IL‐1β, and IL‐6 that were inhibited by lactate in DCs (Figure 4g). Assessment of DC phagocytic capacity using green fluorescent protein‐labeled bacteria revealed that lactate exposure impaired DC phagocytosis, while SnSe partially alleviated this inhibitory effect (Figure 4h,i). Tolerogenic dendritic cells (tDCs) represent a DC subset skewed toward immune tolerance, typically forming in steady‐state or immunosuppressive environments.^[^ ^43^ ^]^ Compared with mature proinflammatory DCs, tDCs exhibit reduced antigen‐presenting capability and preferentially maintain immune tolerance to limit excessive inflammation.^[^ ^40^, ^43^ ^]^ CD63 has been identified as a canonical marker of tDCs, reflecting their immunosuppressive phenotype.^[^ ^40^ ^]^ Flow cytometry analysis showed that lactate treatment significantly increased the proportion of CD11c⁺CD63⁺ tDCs (Figure 4j,k), indicating that lactate drives DCs toward a tolerogenic phenotype and potentially suppresses local inflammatory responses. Notably, the addition of SnSe significantly decreased the proportion of tDCs, suggesting that SnSe can inhibit lactate‐induced conversion of DCs to a tolerogenic state and restore their proinflammatory and antigen‐presenting functions. To further validate the reversal of DC tolerance by SnSe, a secondary LPS stimulation assay was conducted. DCs treated with SnSe produced significantly higher levels of pro‐inflammatory cytokines upon the secondary LPS challenge, indicating functional restoration, whereas control DCs remained tolerant (Figure S16, Supporting Information). These findings highlight a potential strategy for SnSe to reverse lactate‐mediated tolerogenic DC formation, thereby alleviating immunosuppression and enhancing innate immune activity.

In addition, we investigated the functional regulation of BMDMs in a lactate‐enriched environment. Macrophages are key effector cells of the innate immune system, playing critical roles in pathogen phagocytosis, proinflammatory cytokine secretion, and tissue repair modulation.^[^ ^48^, ^49^ ^]^ Their polarization states can be distinguished by surface markers: proinflammatory (M1‐like) macrophages typically exhibit high CD86 and low Arg‐1 expression, favoring inflammatory and bactericidal functions, whereas anti‐inflammatory (M2‐like) macrophages show high CD206 and Arg‐1 expression, promoting inflammation resolution and tissue repair.^[^ ^48^, ^50^ ^]^ Immunofluorescence and flow cytometry analyses revealed that lactate significantly suppressed LPS‐induced proinflammatory polarization of BMDMs, as indicated by decreased CCR7 expression, increased Arg‐1, reduced CD86, and elevated CD206 (Figure 4l,m; Figure S17, Supporting Information), suggesting a shift toward an anti‐inflammatory phenotype. Upon SnSe treatment, BMDM polarization was markedly restored, evidenced by increased CCR7, decreased Arg‐1, elevated CD86, and reduced CD206, indicating enhanced capacity for proinflammatory conversion. These results demonstrate that SnSe, through its lactate dehydrogenase‐mimicking activity, can reverse lactate‐mediated immunosuppression and restore macrophage proinflammatory function. Taken together, SnSe, via its lactate dehydrogenase‐mimicking activity, not only effectively inhibits lactate‐induced formation of tDCs and restores DC maturation, migration, and proinflammatory function, but also reshapes macrophage polarization to enhance their proinflammatory capacity. This suggests that SnSe can broadly improve the lactate‐enriched immunosuppressive microenvironment, thereby enhancing innate immune cell responses to infection or inflammation, and provides a potential approach for targeting metabolic regulation in anti‐biofilm therapy and immune function restoration.

### 2.5 ReSOT Clears Implant‐Associated Biofilms and Reshapes the Local Immune Microenvironment

**Figure 5:**
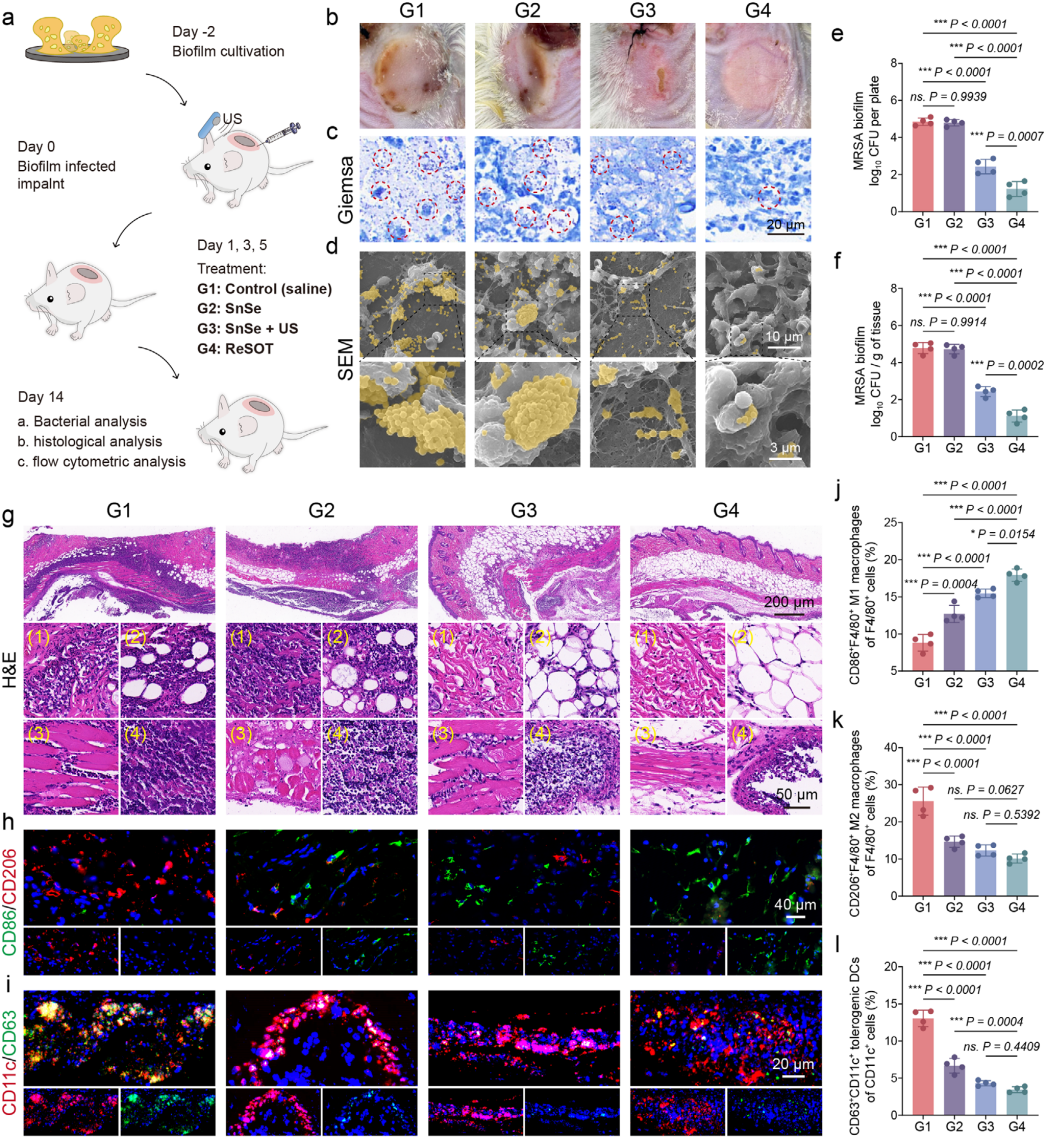
ReSOT clears implant‐associated biofilms and reshapes innate immunity in peri‐implant tissue. a) Schematic diagram of experimental workflow. b) representative digital photographs of MRSA biofilm infected mice. c) Giemsa's staining of peri‐implant tissues. Scale bar, 10 µm. d) SEM images of implants. The yellow color indicates MRSA. Scale bars: 10 µm above and 3 µm below. e) Quantitative analysis of SPC assays from the ultrasonic oscillation solution of the implant, *n* = 4. f) Quantitative analysis of SPC assays from peri‐implant tissue homogenates, *n* = 4. g) H&E staining of peri‐implant tissues. (1) loose connective tissue, (2) subcutaneous fat, (3) superficial muscle, and (4) peri‐implant regions. Scale bar, 200 µm above and 50 µm below. h) Immunofluorescent staining of peri‐implant tissues on day 14. Red pseudo‐color represents CD206, and green pseudo‐color represents CD86. Scale bar, 40 µm. i) Immunofluorescent staining of peri‐implant tissues on day 14. Red pseudo‐color represents CD11c, and green pseudo‐color represents CD63. Scale bar,240 µm. j) Flow cytometric analysis of CD45^+^CD11b^+^F4/80^+^CD86^+^ M1 macrophages on day 14, *n* = 4. k) Flow cytometric analysis of CD45^+^CD11b^+^F4/80^+^CD206^+^ M2 macrophages on day 14, *n* = 4. l) Flow cytometric analysis of CD45^+^CD11c^+^CD63^+^ tDCs on day 14, *n* = 4. ^*^ *p* < 0.05, ^**^ *p* < 0.01, ^***^ *p* < 0.001. Data are means ± SD.

Given the excellent anti‐biofilm activity of ReSOT observed in vitro and the intrinsic capacity of SnSe to reverse lactate‐mediated immunosuppression, we further evaluated the therapeutic efficacy and immunomodulatory effects of ReSOT in a murine model of MRSA biofilm infection on implanted devices. The experimental procedure is illustrated in **Figure** **5**a. Polyether ether ketone (PEEK) implants were incubated in a suspension of MRSA at 10^9^ CFU/mL for two days to allow biofilm formation. On day 0, all BALB/c mice received subcutaneous implantation of the pre‐incubated PEEK implants and were then randomly assigned into 4 groups according to the subsequent treatment regimen: control (saline), SnSe alone, SnSe + US, and ReSOT. Treatments were administered on days 1, 3, and 5. To further elucidate the in vivo mechanism of ReSOT, lactate levels in biofilm‐surrounding tissues were quantified and compared with those in normal tissues. Lactate concentrations were markedly elevated in tissues adjacent to biofilms, indicating a high‐lactate microenvironment and supporting the role of ReSOT in modulating lactate‐related metabolic pathways during antibacterial activity. Furthermore, SnSe treatment substantially reduced lactate levels in biofilm‐surrounding tissues, suggesting that ReSOT can reshape the local metabolic microenvironment, which may further enhance biofilm disruption as well as the restoration of immune function (Figure S18, Supporting Information). Hematoxylin and Eosin (H&E) staining revealed no obvious abnormalities in major organs (Figure S19, Supporting Information). Gross observation on day 14 revealed severe tissue damage in the control and SnSe‐alone groups, while the SnSe + US group exhibited notably reduced inflammation. Remarkably, the ReSOT group showed near‐complete tissue restoration, with the implantation sites covered by intact skin (Figure 5b). Giemsa staining showed abundant dense bacterial clusters in the control and SnSe groups, whereas bacterial load was markedly reduced in the SnSe + US group and nearly completely cleared in the ReSOT group (Figure 5c). SEM further confirmed these findings: thick biofilm structures were observed on the implant surfaces of the control and SnSe groups, only sparse bacterial residues remained in the SnSe + US group, and the ReSOT group displayed virtually no formed biofilm (Figure 5d). Consistently, quantitative colony counting demonstrated that SnSe + US significantly reduced bacterial burden on the implants and surrounding tissues, while ReSOT exhibited the most pronounced anti‐biofilm effect (Figure 5e,f).

H&E staining further corroborated the observations regarding infection containment and tissue protection (Figure 5g). In the control and SnSe groups, extensive tissue necrosis and inflammatory infiltration were evident, affecting loose connective tissue, subcutaneous fat, superficial muscle, and peri‐implant regions. Compared with these groups, the SnSe + US group showed milder infection involvement, although localized abscesses were still present around the implants. In contrast, the ReSOT group exhibited near‐normal tissue architecture, indicating the superior capacity of ReSOT to control biofilm‐associated infection. To elucidate the role of immune modulation in infection resolution, we analyzed the phenotypes of immune cells in the peri‐implant tissues. Immunofluorescence results showed that the peri‐implant regions of the control group were predominantly populated by M2 macrophages, whereas the other three groups exhibited notable distributions of M1 macrophages (Figure 5h). Furthermore, DC phenotyping revealed enrichment of tDCs in the control group, while their prevalence was markedly reduced in the remaining groups (Figure 5i). Flow cytometry analyses further supported these observations, demonstrating that SnSe treatment significantly increased the distribution of M1 macrophages (Figure 5j; Figure S20, Supporting Information) in the peri‐implant tissues while reducing immunosuppressive cell populations, including M2 macrophages (Figure 5k; Figure S21, Supporting Information) and tolerogenic DCs (Figure 5l; Figure S22, Supporting Information). These findings suggest that SnSe has the potential to reshape the local immune microenvironment.

**Figure 6:**
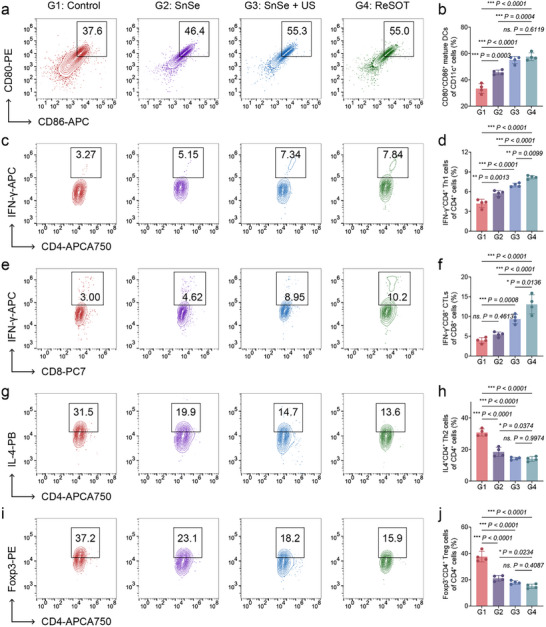
ReSOT reshapes adaptive immunity in peri‐implant tissue. a) Flow cytometric analysis and b) corresponding quantitative analysis of CD45^+^CD11c^+^CD80^+^CD86^+^ mature DCs on day 14, *n* = 4. c) Flow cytometric analysis and d) corresponding quantitative analysis of CD45^+^CD3^+^CD4^+^IFN‐γ^+^ Th1 cells on day 14, *n* = 4. e) Flow cytometric analysis and f) corresponding quantitative analysis of CD45^+^CD3^+^CD8^+^IFN‐γ^+^ CTLs on day 14, *n* = 4. g) Flow cytometric analysis and h) corresponding quantitative analysis of CD45^+^CD3^+^CD4^+^IL‐4^+^ Th2 cells on day 14, *n* = 4. i) Flow cytometric analysis and j) corresponding quantitative analysis of CD45^+^CD3^+^CD4^+^Foxp3^+^ Tregs on day 14, *n* = 4. ^*^ *p* < 0.05, ^**^ *p* < 0.01, ^***^ *p* < 0.001. Data are means ± SD.

Notably, as key antigen‐presenting cells, DCs not only capture and process pathogens in innate immunity but also relay antigenic information to T cells via co‐stimulatory molecules and chemokine receptors, thereby bridging innate and adaptive immune responses.^[^ ^45^, ^46^, ^47^ ^]^ Evaluation of DC maturation revealed that SnSe treatment significantly increased CD80 and CD86 expression, indicating restored DC activation capacity (**Figure** **6**a,b). Since SnSe reduces the proportion of tolerogenic DCs and promotes DC maturation in peri‐implant tissues, which may further enhance adaptive immunity, we further analyzed the local adaptive immune microenvironment. Flow cytometry analysis revealed that the proportions of Th1 cells and cytotoxic T lymphocytes (CTLs) were low in the control group (Figure 6c–f), whereas Th2 cells and Tregs in the peri‐implant tissue were relatively abundant (Figure 6g–j). Following SnSe treatment, the frequencies of Th1 cells and CTLs were significantly elevated, while Th2 and Treg populations decreased, indicating a shift in the local adaptive immune response from an immunosuppressive state toward a pro‐inflammatory, infection‐clearing profile. Importantly, ReSOT not only maximally suppressed the formation of tDCs but also amplified DC‐mediated T cell activation, resulting in the most pronounced Th1/CD8⁺ T cell responses while effectively reducing immunosuppressive Treg and Th2 cells (Figure 6c–j). Taken together, ReSOT not only achieves efficient clearance of biofilm on implant surfaces and surrounding tissues through its intrinsic anti‐biofilm activity but also synergistically enhances the functions of innate and adaptive immune cells, remodeling the local immune microenvironment to enable comprehensive infection control and effective restoration of tissue function.

## 3 Discussion

In conclusion, redox homeostasis within bacterial biofilms has emerged as a critical determinant of resistance to both antibiotics and nanocatalytic therapies, yet it remains an underexplored therapeutic target. In this work, we propose for the first time a redox stress oscillation therapy, ReSOT, that synergistically integrates enzyme‐like catalysis and piezoelectric activation to achieve dynamic modulation of bacterial redox balance. By exploiting the lactate dehydrogenase‐mimicking activity of SnSe nanosheets to induce reductive stress, followed by US‐triggered piezoelectric catalysis to impose oxidative stress, this strategy establishes a continuous oscillatory redox pressure that effectively disrupts biofilm integrity and overcomes the intrinsic adaptive resistance of bacteria.

Beyond its potent antibiofilm efficacy, ReSOT also alleviates lactate‐driven immunosuppression within the infection microenvironment, restoring the functional activity of both innate and adaptive immune cells and promoting a pro‐inflammatory and pathogen‐clearing immune profile. This dual action of simultaneous biofilm eradication and immune microenvironment remodeling highlights the strong therapeutic advantage and clinical relevance of this platform, particularly for refractory implant‐associated infections. Importantly, although ReSOT is demonstrated here in the context of bacterial biofilm infections, its mechanistic basis suggests broader applicability. The capability to regulate metabolic redox states and reshape immunosuppressive microenvironments indicates promising potential for extension to other pathological conditions characterized by dysregulated redox balance and excessive lactate accumulation, such as solid tumors, chronic inflammatory diseases, and ischemia‐related disorders. In these contexts, SnSe‐driven ReSOT may offer a versatile strategy for coordinated metabolic intervention and immune reactivation.

Overall, this work establishes ReSOT as a conceptually innovative and translatable therapeutic paradigm, opening new avenues for the development of nanozyme‐based redox medicine. These findings may inspire future exploration of oscillatory redox‐regulating platforms across diverse biomedical applications and contribute to reshaping therapeutic strategies for overcoming resistance in complex disease microenvironments.

## 4 Experimental Section

### Statistical Analysis

The data are presented as mean ± s.d. unless otherwise stated, and “n” refers to the number of biological replicates. All data were analyzed in GraphPad Prism 10 (La Jolla, CA, USA) using one‐way ANOVA and Student's t‐test. Significance levels are denoted as ^*^ *p* < 0.05, ^**^ *p* < 0.01, ^***^ *p* < 0.001, ^****^ *p* < 0.0001. The data are expressed as means ± standard deviation (SD).

### Animal Ethics Statement

All animal experimental procedures were in accordance with the institutional policies and federal regulations established by the Institutional Animal Care and Use Committee of the Shanghai Sixth People's Hospital. The experimental protocols were approved by Shanghai Sixth People's Hospital Affiliated to Shanghai Jiao Tong University School of Medicine, with the official registration number DWLL2025‐0983. BALB/c mice (6–8 weeks old) were anesthetized, and PEEK implants pre‐coated with MRSA biofilms were subcutaneously implanted on the dorsal region. Mice were randomly assigned to four groups: control (saline), SnSe, SnSe + US, and ReSOT. Treatments were applied on days 1, 3, and 5. Mice were monitored for 14 days. On day 14, peri‐implant tissues and implants were collected for bacterial analysis, pathological analysis and flow cytometry analysis.

### In Vivo MRSA Biofilm Implantation Model

All animal experiments were approved by Shanghai Sixth People's Hospital Affiliated to Shanghai Jiao Tong University School of Medicine. For pathological analysis, peri‐implant tissues were harvested, fixed, and processed for H&E staining to assess tissue architecture, necrosis, and inflammatory infiltration. Immunofluorescence staining was performed to evaluate the distribution of M1 and M2 macrophages and tDCs. Major organs such as the heart, liver, spleen, lungs, and kidneys were assessed for the biosafety of SnSe using H&E staining. For flow cytometry analysis, peri‐implant tissues were digested to obtain single‐cell suspensions using Liberase^TM^ TL (Roche, Switzerland) and DNase‐I (MedChemExpress, USA). Detailed information on the antibodies applied is listed in Table S2 (Supporting Information). A complete gating strategy, including FSC/SSC selection, dead cell exclusion, and subset‐specific markers, was applied for the analysis of in vivo flow cytometry data (Figures S23 and S24, Supporting Information). Intracellular staining for cytokines and transcription factors was performed using fixation/permeabilization buffers (Thermo Fisher Scientific, USA). Data were acquired on the flow cytometer (Beckman Coulter, USA) and analyzed with FlowJo v10.

## Conflict of Interest

The authors declare no conflict of interest.

## Author Contributions

M.G., Z.G., and H.L. designated the idea of this work. M.G., Z. G., Z.Z., Z.R., T.S., and L.L. performed the experiments. M.G. and Z.G. wrote the initial manuscript draft., Y.C., Z.G., J.H., C.T., and H.L. revised the manuscript, supervised the project and commented on it. All of the authors contributed to discussing the results and implications, and editing the manuscript.

## Supporting information



Supporting Information

## Data Availability

The data that support the findings of this study are available from the corresponding author upon reasonable request.
