# Supplementary material for: An Enzyme‐Like Catalyzed Nanosheets for Redox Stress Oscillation Therapy Against Bacterial Infections
Source: Adv Sci (Weinh). 2025 Dec 17;13(18):e19334. doi: 10.1002/advs.202519334 (PMC13042778; doi:10.1002/advs.202519334)
Supplement: Supplementary file 1 — Supporting Information [file ADVS-13-e19334-s001.docx]

**Supporting Information**

**An Enzyme-Like Catalyzed Nanosheets for Redox Stress Oscillation Therapy Against Bacterial Infections**

*Min Ge, Zhao Guo*,* *Zhiming Zhang,* *Lanlu Lu, Zesong Ruan, Tingwang Shi, Yunfeng Chen, Ju Huang*, Chaoliang Tan, Han Lin**

**The PDF file includes:**

Methods

Supporting Figure S1-S24

Supporting Tables S1 to S2

**Methods**

**Characterization**

Transmission electron microscope (TEM) and high-resolution transmission electron microscope (HRTEM) photographs were acquired on a JEM-F200 transmission electron microscope (JEOL, Japan). X-ray diffraction (XRD) was measured on a D2 Phaser XRD system (Bruker, Germany). X-ray photoelectron spectroscopy (XPS) was performed on a Thermo Scientific K-Alpha (Thermo, US). Atomic force microscope (AFM) analysis was carried out on Bruker Dimension Icon system (Bruker, Germany). UV-vis-NIR absorption spectra were conducted on UV-3600i Plus Shimadzu UV-vis-NIR spectrometer.

**Preparation of SnSe Nanosheets**

The SnSe compound with a two-dimensional structure was synthesized via an improved co-deposition precipitation method based on previous reports. Briefly, stoichiometric NaOH (6.4 g) and Se powder (40 mg) were dissolved in 16 mL of deionized water under vigorous stirring, noted as the Se solution. Concurrently, citric acid (8 mg) and SnCl₂·2H₂O (0.36 mg) were dissolved in 16 mL of deionized water under gentle stirring to prepare the Sn solution. Subsequently, the Sn solution was slowly added dropwise to the Se solution under vigorous stirring to obtain SnSe nanosheets. The product was collected by centrifugation at 13000 rpm, sequentially washed with ethanol and deionized water, dried, and stored for later use.

**The XAFS Measurements and Analysis**

The XAFS data at the Se k-edge of the samples were collected at the beam line BL17B1 of the Shanghai Synchrotron Radiation Facility (SSRF) at room temperature. Data reduction, data analysis, and EXAFS fitting were performed and analyzed with the Athena and Artemis programs of the Demeter data analysis packages that utilizes the FEFF6 program to fit the EXAFS data. The energy calibration of the sample was conducted through standard Se foil, which as a reference was simultaneously measured. A linear function was subtracted from the pre-edge region, then the edge jump was normalized using Athena software. The χ(k) data were isolated by subtracting a smooth, third-order polynomial approximating the absorption background of an isolated atom. The k^3^-weighted χ(k) data were Fourier transformed after applying a Hanning window function (Δk = 1.0). For EXAFS modeling, the global amplitude EXAFS (CN, R, σ^2^ and ΔE_0_) were obtained by nonlinear fitting, with least-squares refinement, of the EXAFS equation to the Fourier-transformed data in R-space, using Artemis software, EXAFS of the Se foil was fitted and the obtained amplitude reduction factor S_0_^2^ value was set in the EXAFS analysis to determine the coordination numbers in sample.

**LDH-Mimetic Activity Assay of SnSe**

SnSe nanosheets were diluted to the desired concentrations (50, 100, and 200 μg/ml) in the buffer recommended by the LDH assay kit (MAK066). In a 96-well plate, 200 μL of the LDH substrate solution was mixed with the diluted SnSe samples, and blank controls containing only substrate were included. The plate was incubated at 37 °C, and absorbance at 490 nm was measured every 10 minutes using a microplate reader (Epoch BioTEK, USA).

**Cell Viability Assay**

The cytotoxicity of SnSe was evaluated using the cck-8 assay. Briefly, L929 cells and bone marrow-derived macrophages (BMDMs) were seeded in 96-well plates (50,000 cells/mL) and treated with various concentrations of SnSe (0-1000 μg/ml) for 24 hours. After 24h, the medium was withdrawn and the cells were incubated with 10% cck8 kit for 2h. The absorbance of each sample at 450 nm was measured using a microplate reader (Epoch BioTEK, USA). The cell viability (%) was evaluated based on the following equation:

*Cell Viability (%) = (ODs - ODc)/ODc,*

where ODs and ODc indicate the optical density (OD) value of the sample and control, respectively. Based on these results, 100 μg/ml SnSe was selected for subsequent experiments.

**Biofilm Formation and ReSOT Treatment**

Before the experiment, frozen cultures of methicillin-resistant *Staphylococcus aureus* (MRSA, ATCC 43300) or *Escherichia coli* (*E. coli,* ATCC 35218) were activated by overnight incubation in TSBG. The bacterial concentration was adjusted to 0.5 McFarland standard, corresponding to approximately 10^8^ CFU/ml, and then further diluted to 10^6^ CFU/ml with TSBG. The bacterial suspension was added to sterile experimental substrates, including titanium discs, 12-well plates, and confocal dishes. Biofilms were allowed to form by incubating the samples at 37°C for 48 hours, after which planktonic bacteria were removed by washing three times with PBS to obtain mature biofilms. For treatment, 1 ml of TSBG, or 1 ml of SnSe (100 μg/ml, resuspended in TSBG) was added to replace the biofilm supernatant, with or without ultrasonic stimulation (1.0 W·cm⁻², 50% duty cycle, 10 minutes), and samples were further incubated for 8 hours. For the Redox Oscillation Therapy (ReSOT), bacteria were first co-cultured with SnSe for 4 hours, followed by 10 minutes of ultrasonic treatment, and then further incubated for an additional 4 hours (with SnSe concentration and ultrasound conditions consistent with the other groups).

Colony-forming units (CFUs) were quantified using the spread plate counting assay. Treated biofilms were collected, serially diluted, and plated evenly on sheep blood agar plates. After incubation at 37°C for 18 hours, colonies were photographed and counted. For crystal violet staining, biofilms were fixed with 4% paraformaldehyde for 30 minutes, washed three times with PBS, and stained with crystal violet for 10 minutes. Excess stain was removed by washing with PBS three times, followed by photography. The crystal violet was then eluted using 33% acetic acid, and absorbance at 595 nm was measured with a microplate reader. For live/dead staining, biofilms were collected and stained with Syto9/PI (1:1000) for 30 minutes, and the stained biofilms were observed under a confocal laser scanning microscope to assess biofilm structure and bacterial viability.

**Non-targeted Metabolomics of MRSA**

MRSA was cultured in tryptic soy broth supplemented with glucose (TSBG) with or without SnSe (100 μg/ml), and bacterial pellets were collected for non-targeted metabolomics analysis. Metabolites were extracted, derivatized, and analyzed using liquid chromatography–mass spectrometry (LC-MS). Principal component analysis (PCA) was performed to assess global metabolic alterations. Differential metabolites were identified based on fold change and statistical significance, and KEGG pathway enrichment analysis was conducted to reveal affected metabolic pathways.

**Bone Marrow-derived Dendritic Cells (BMDCs) and Macrophages (BMDMs) Culture**

Bone marrow cells were harvested from femurs and tibias of BALB/c mice and cultured in RPMI 1640 medium supplemented with GM-CSF (20 ng/mL) and IL-4 (10 ng/mL) for BMDC differentiation or with M-CSF (20 ng/mL) for BMDM differentiation. Cells were treated with LPS (100 ng/mL) in the presence or absence of SnSe and/or lactate (5 mmol/L).

**Flow Cytometry Analysis in vitro**

For BMDCs, surface expression of maturation markers CD80, CD86, CD40, CD83, and chemokine receptor CCR7 was assessed. Tolerogenic DCs (tDCs) were identified as CD11c⁺CD63⁺ cells. For BMDMs, polarization markers CD86 and CD206 were evaluated. Cells were stained with fluorophore-conjugated antibodies according to standard protocols and analyzed using flow cytometry. Detailed information on the antibodies applied is listed in **Table S2**.

**Cytokine Secretion Assay**

Cytokine levels (TNF-α, IL-1β, IL-6) in BMDC culture supernatants were measured using ELISA kits following the manufacturer’s instructions.

**Phagocytosis Assay**

BMDCs were incubated with GFP-labeled bacteria at a 1:100 phagocyte-to-target ratio for 2 hours at 37°C. Cells were washed, harvested, and analyzed by flow cytometry to quantify GFP uptake as a measure of phagocytic capacity.

**Supporting Figures**

**
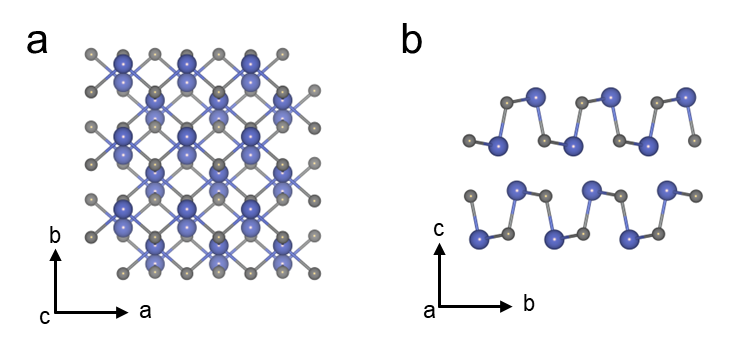
**

**Figure S1.** Atomic structure model of SnSe projected on the (a) c-axis and (b) a-axis.


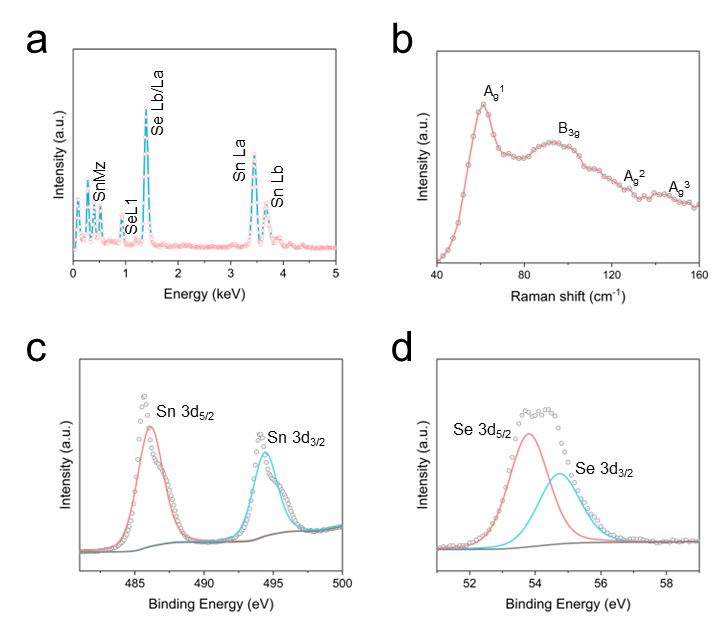


**Figure S2.** (a) The Energy-dispersive spectrum (EDS) of SnSe NSs. (b) The Raman spectrum of a SnSe NSs. High-resolution XPS spectra of (c) Sn 3d and (d) Se 3d.


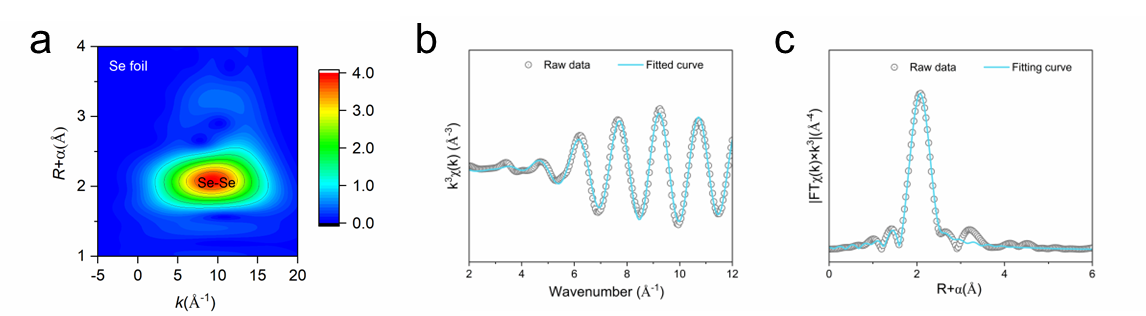


**Figure S3.** (a) Wavelet transform images for the EXAFS signals of Se foil. (b) The Se k-edge EXAFS fitting result of Se foil at k space. c) EXAFS fitting result of Se foil in R space.


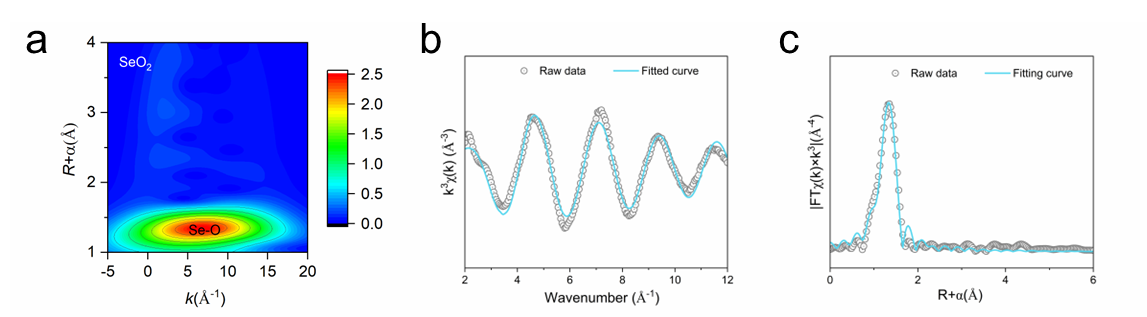


**Figure S4.** (a) Wavelet transform images for the EXAFS signals of SeO_2_. (b) The Se k-edge EXAFS fitting result of SeO_2_ at k space. c) EXAFS fitting result of SeO_2_ in R space.

**
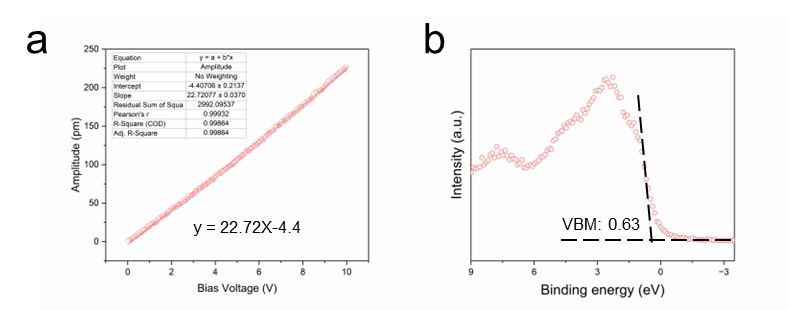
**

**Figure S5.** (a) Calculation fitting curve of the piezoelectric constant d_33_ of SnSe NSs. (b) XPS Valence band spectrum analysis of SnSe NSs.


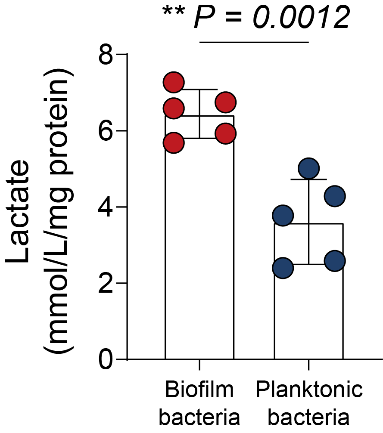


**Figure S6.** Lactate concentrations in the supernatants of biofilm and planktonic bacterial cultures, n = 5.

**
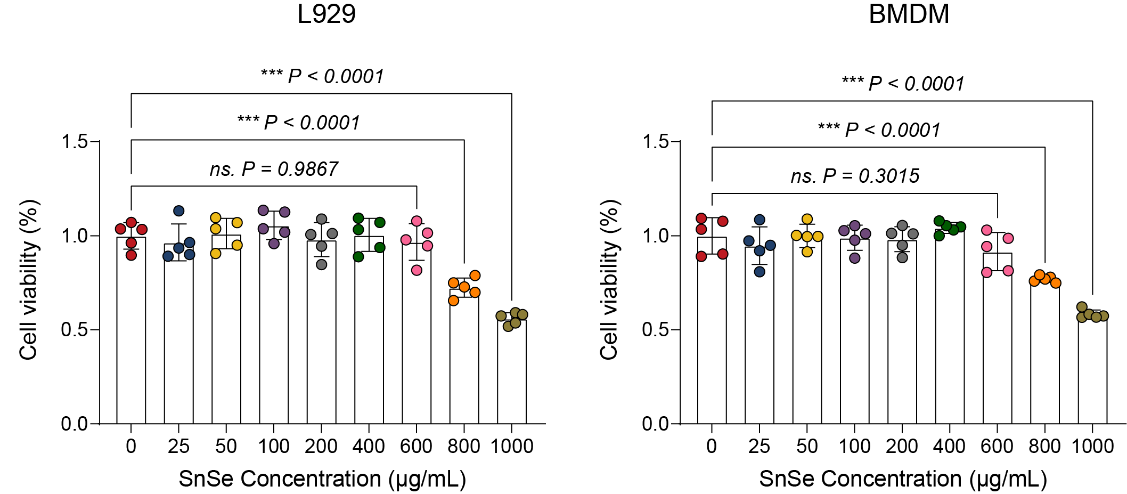
**

**Figure S7.** Cell viability of L929 and BMDMs co-cultured with different concentrations of SnSe, n = 5.

**
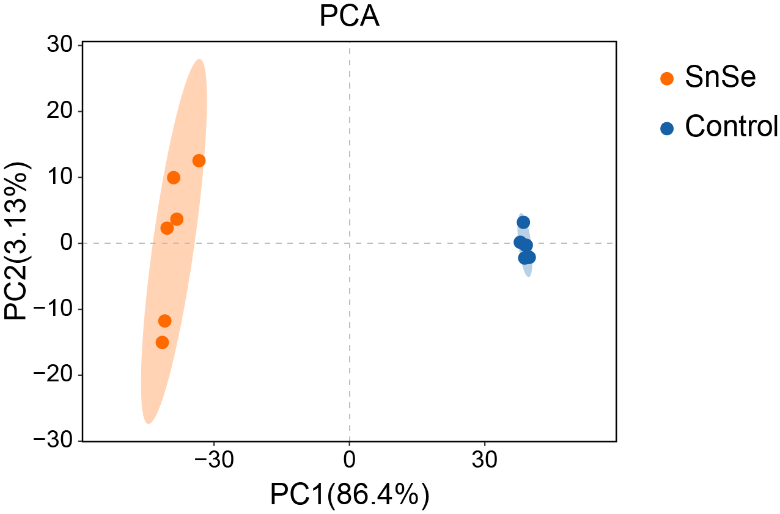
**

**Figure S8.** PCA analysis of differential metabolites.

**
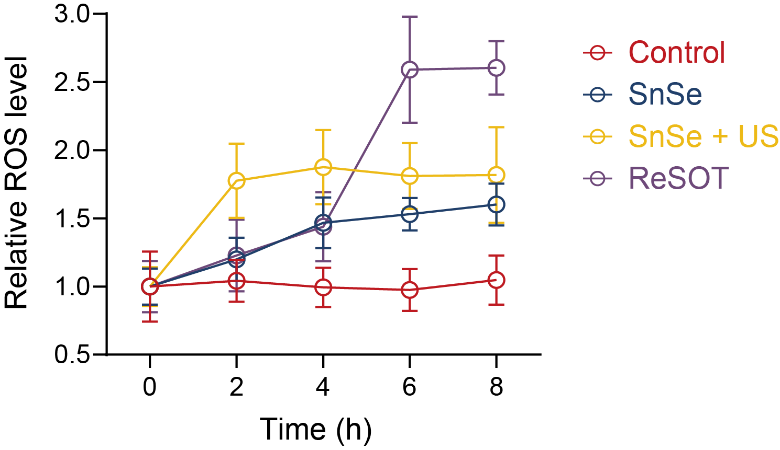
**

**Figure S9.** Time-course of intracellular ROS level in MRSA biofilm following various intervention, n = 6.

**
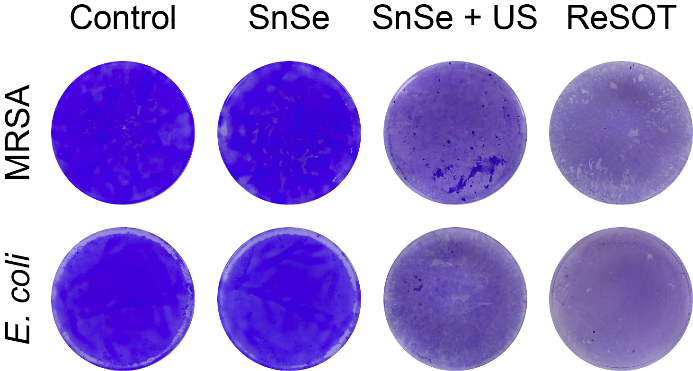
**

**Figure S10.** Representative images of biofilms stained by crystal violet from MRSA and *E. coli* after various treatments.


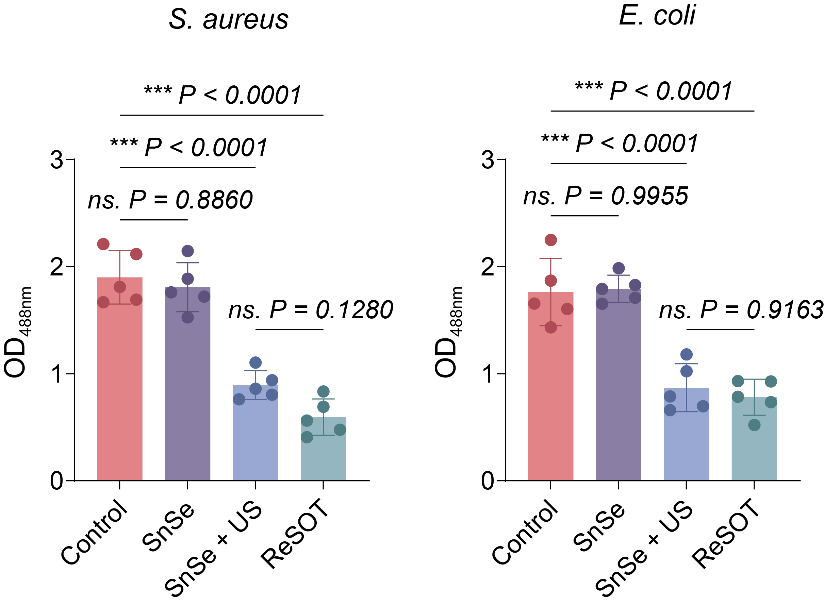


**Figure S11.** Biofilm polysaccharide content of MRSA and *E. coli* biofilms after various treatments, n = 5.

**
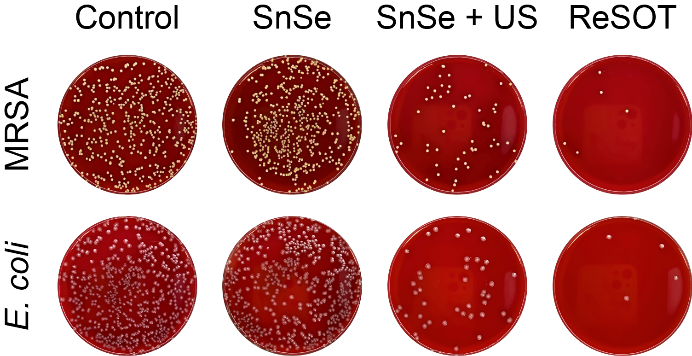
**

**Figure S12.** Representative images of SPCM from planktonic MRSA and *E. coli* after various treatments.


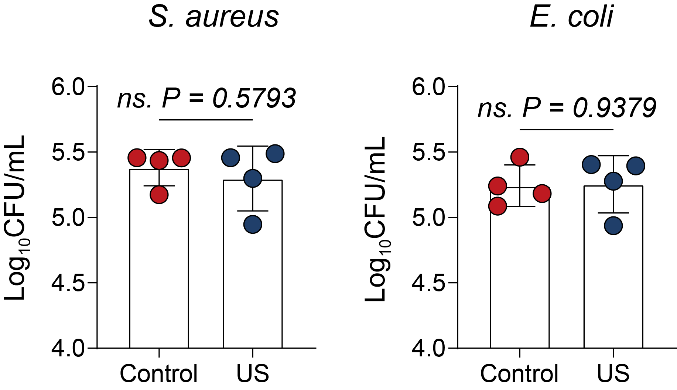


**Figure S13.** CFU counts verifying the role of ultrasound-only, n = 4.


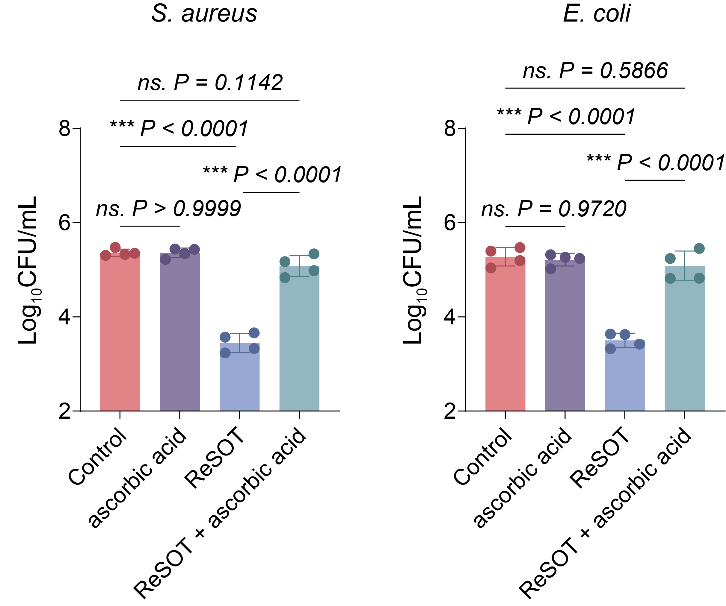


**Figure S14.** CFU counts verifying the role of ROS in biofilm clearance, with ascorbic acid applied as a ROS scavenger, n = 4.

**
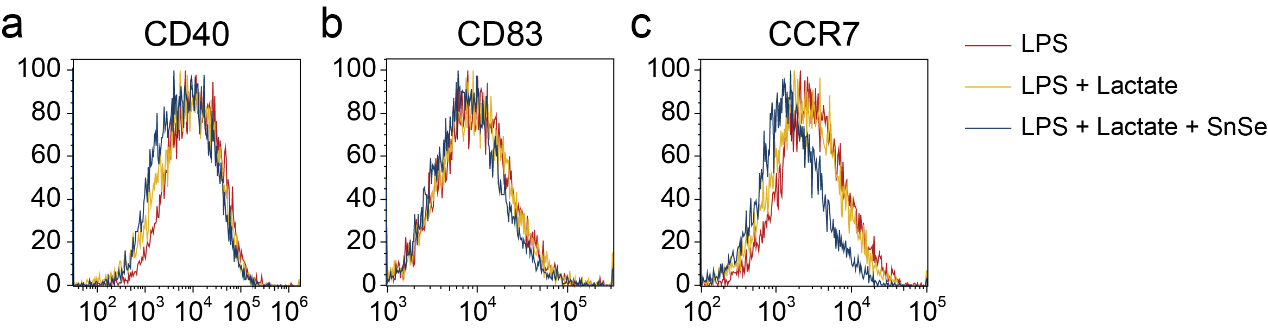
**

**Figure S15.** Flow cytometric analysis of CD40 (a) and CD83 (b) and CCR7 (c) expression in BMDCs after various treatments.


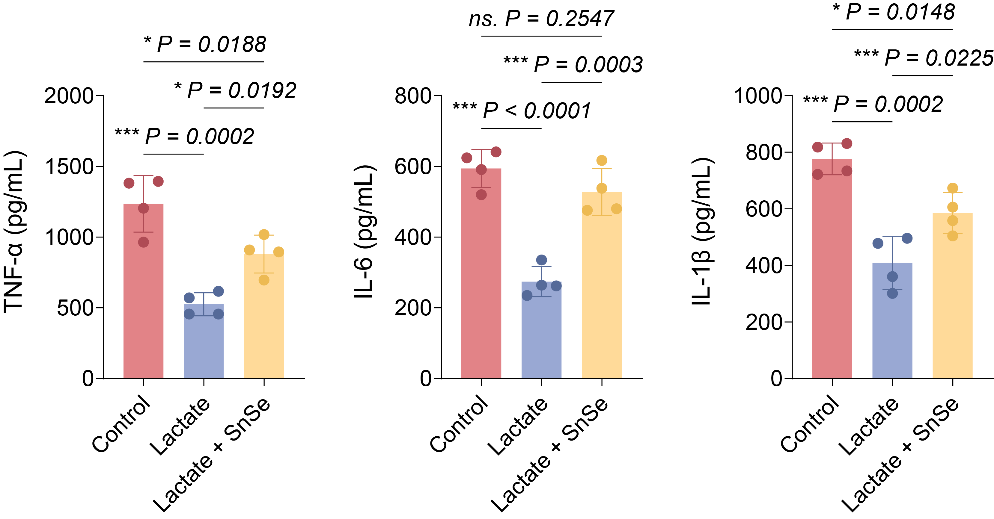


**Figure S16.** TNF-α, IL-6 and IL-1β secreted by BMDCs after secondary LPS exposure following different treatments, n = 4.

**
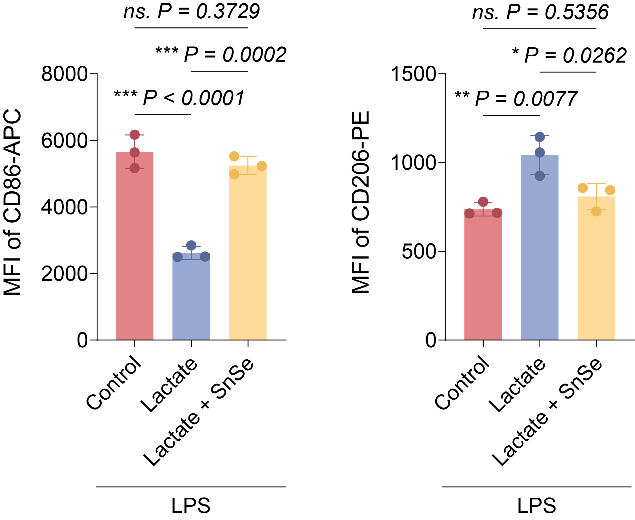
**

**Figure S17.** Quantitative analysis of CD86 and CD206 MFI in BMDMs detected by flow cytometry, n = 3.


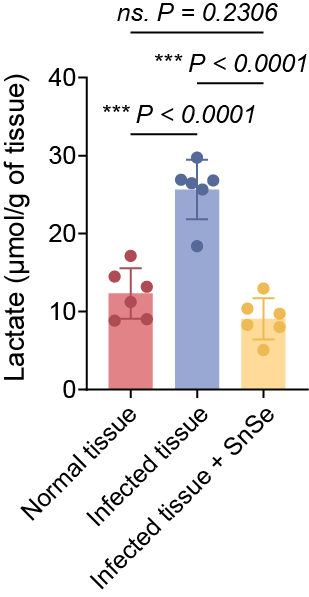


**Figure S18.** Lactate concentrations in normal tissues, infected tissues, and infected tissues treated with SnSe, n = 6.

**
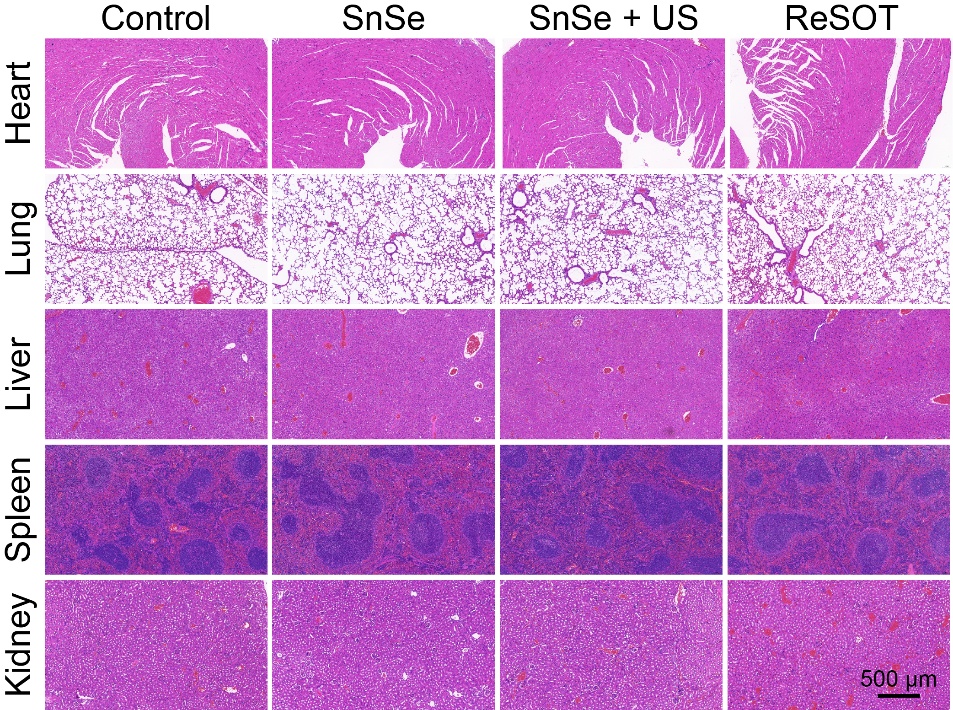
**

**Figure S19.** H&E staining of vital organs from implant associated biofilm infection model mice of each treatment group. Scale bar, 500 μm.

**
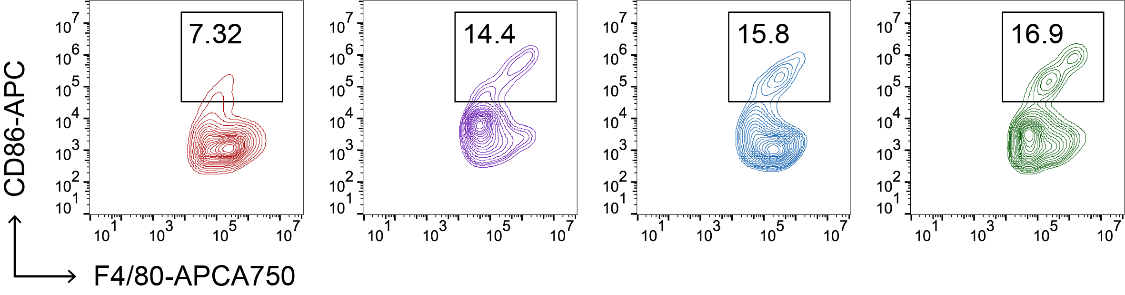
**

**Figure S20.** Flow cytometric analysis of CD45^+^CD11b^+^F4/80^+^CD86^+^ M1 macrophages.

**
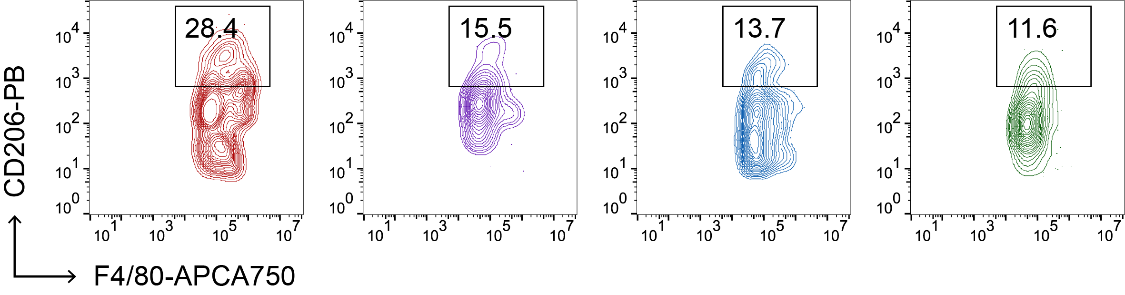
**

**Figure S21.** Flow cytometric analysis of CD45^+^CD11b^+^F4/80^+^CD206^+^ M2 macrophages.

**
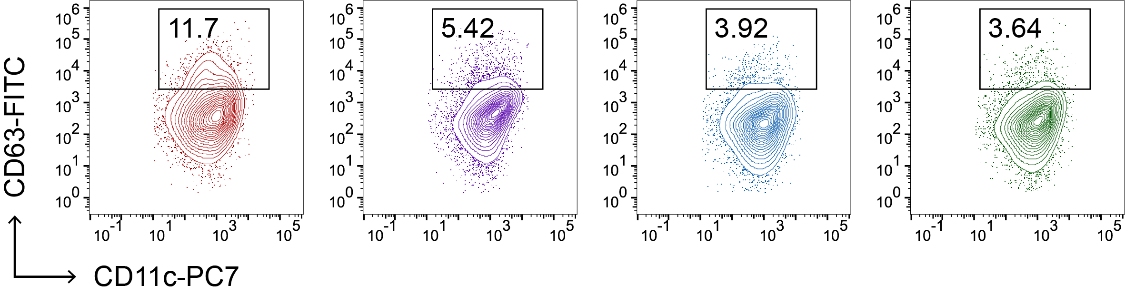
**

**Figure S22.** Flow cytometric analysis of CD45^+^CD11c^+^CD63^+^ tDCs.


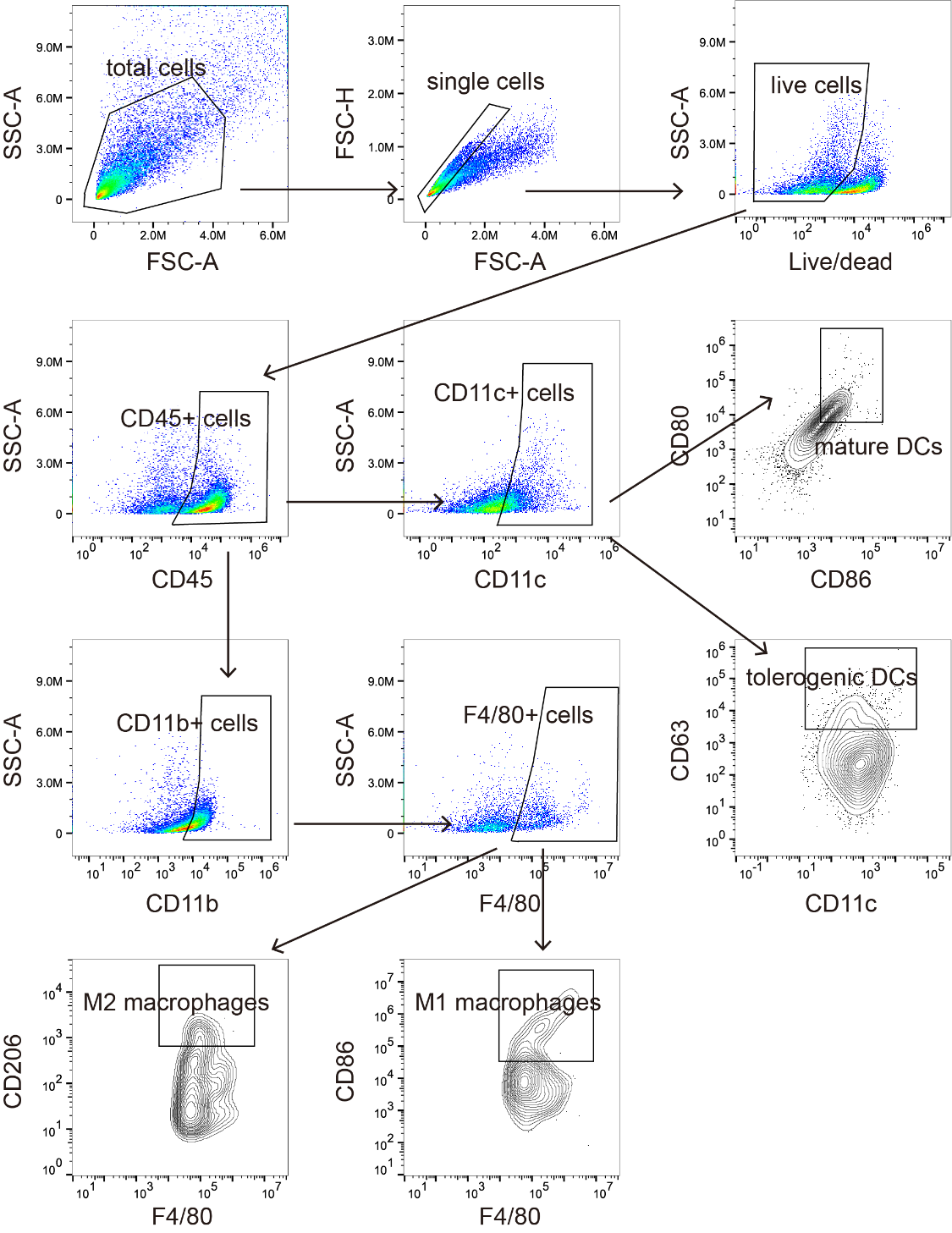


**Figure S23.** Gating strategy for M1 macrophages, M2 macrophages, mature DCs and tolerogenic DCs from murine model of MRSA biofilm infection on implanted devices.


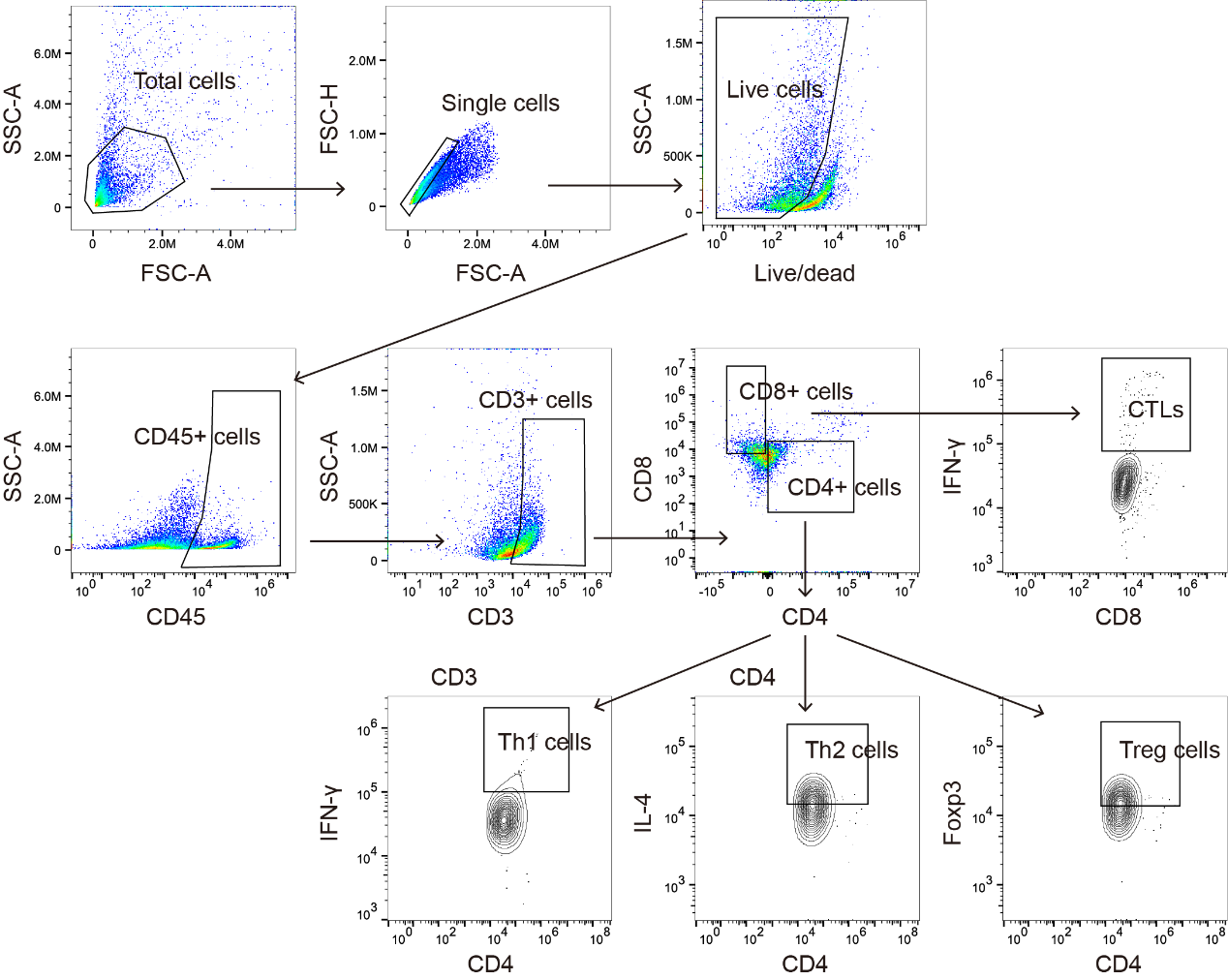


**Figure S24.** Gating strategy for Th1, Th2, Treg and CTL cells from murine model of MRSA biofilm infection on implanted devices.

**Supporting Tables**

**Table S1.** EXAFS fitting parameters at the Se *K*-edge for various samples.

| Sample | Shell | CN^a^ | R(Å)^b^ | σ^2^(Å^2^)^c^ | ΔE_0_(eV)^d^ | K-range/Å^-1^ | R-range/Å | R factor |
| --- | --- | --- | --- | --- | --- | --- | --- | --- |
| Se foil | Se-Se | 2.0* | 2.38±0.01 | 0.0039±0.0007 | 7.0±0.7 | 2.0-13.0 | 1.0-2.6 | 0.0167 |
| SeO_2_ | Se-O | 2.0±0.2 | 1.72±0.01 | 0.0030±0.0011 | 13.0±0.8 | 2.0-13.0 | 1.0-1.8 | 0.0083 |
| SnSe | Se-Se | 0.2±0.1 | 2.34±0.01 | 0.0008±0.0017 | 6.0±0.3 | 3.0-13.0 | 1.6-3.0 | 0.0055 |
|  | Se-Sn | 2.0±0.1 | 2.76±0.01 | 0.0095±0.0008 |  |  |  |  |

*^a^CN*, coordination number; *^b^R*, the distance between absorber and backscatter atoms; *^c^σ*^2^, Debye-Waller factor , Debye-Waller factor to account for both thermal and structural disorders; *^d^ΔE*_0_, inner potential correction; *R* factor indicates the goodness of the fit. *S*_0_^2^ was fixed to 0.998, accourding to the experimental EXAFS fit of Se foil by fixing *CN* as the known crystallographic value. *This value was fixed during EXAFS fitting. Error bounds that characterize the structural parameters obtained by EXAFS spectroscopy were estimated as CN ± 20%; R ± 1%; σ2 ± 20%; ΔE0 ± 20%. A reasonable range of EXAFS fitting parameters: 0.700 < *Ѕ*_0_^2^ < 1.000; *CN >* 0; *σ*^2^ > 0 Å^2^; |Δ*E*_0_| < 15 eV; *R* factor < 0.02.

**Table S2.** Antibody list for flow cytometry.

| Antibody | Color | Clone | Company (Cat.#) | Dilution |
| --- | --- | --- | --- | --- |
| CD45 | PerCP/Cyanine5.5 | 30-F11 | Biolegend (103132) | 1:100 |
| CD11c | FITC | N418 | Biolegend (117305) | 1:100 |
| CD80 | PE | 16-10A1 | Biolegend (104707) | 1:100 |
| CD86 | APC | GL-1 | Biolegend (105011) | 1:100 |
| CD11b | APC/Fire™ 750 | M1/70 | Biolegend (101261) | 1:100 |
| CCR7 | PE/Cyanine7 | AB12 | Biolegend (120123) | 1:100 |
| CD40 | PE | FGK45 | Biolegend (157506) | 1:100 |
| CD83 | APC | Michel-19 | Biolegend (121510) | 1:100 |
| CD63 | PE/Cyanine7 | NVG-2 | Biolegend (143910) | 1:100 |
| F4/80 | Brilliant Violet 785™ | BM8 | Biolegend (123141) | 1:100 |
| CD206 | Brilliant Violet 421™ | C068C2 | Biolegend (141717) | 1:100 |
| CD3 | FITC | 145-2C11 | Biolegend (100305) | 1:100 |
| CD4 | APC/Fire™ 750 | RM4-5 | Biolegend (100567) | 1:100 |
| CD8a | PE/Cyanine7 | 53-6.7 | Biolegend (100721) | 1:100 |
| IFN-γ | APC | XMG1.2 | Biolegend (505809) | 1:100 |
| IL-4 | Brilliant Violet 421™ | 11B11 | Biolegend (504119) | 1:100 |
| Foxp3 | PE | 150D | Biolegend (320008) | 1:100 |
